# Supplementary figures and images for: Post-transcriptional suppression of the pioneer factor Zelda protects the adult Drosophila testis from activation of the ovary program
Source: PLoS Biol. 2025 Dec 18;23(12):e3003535. doi: 10.1371/journal.pbio.3003535 (PMC12714197; doi:10.1371/journal.pbio.3003535)

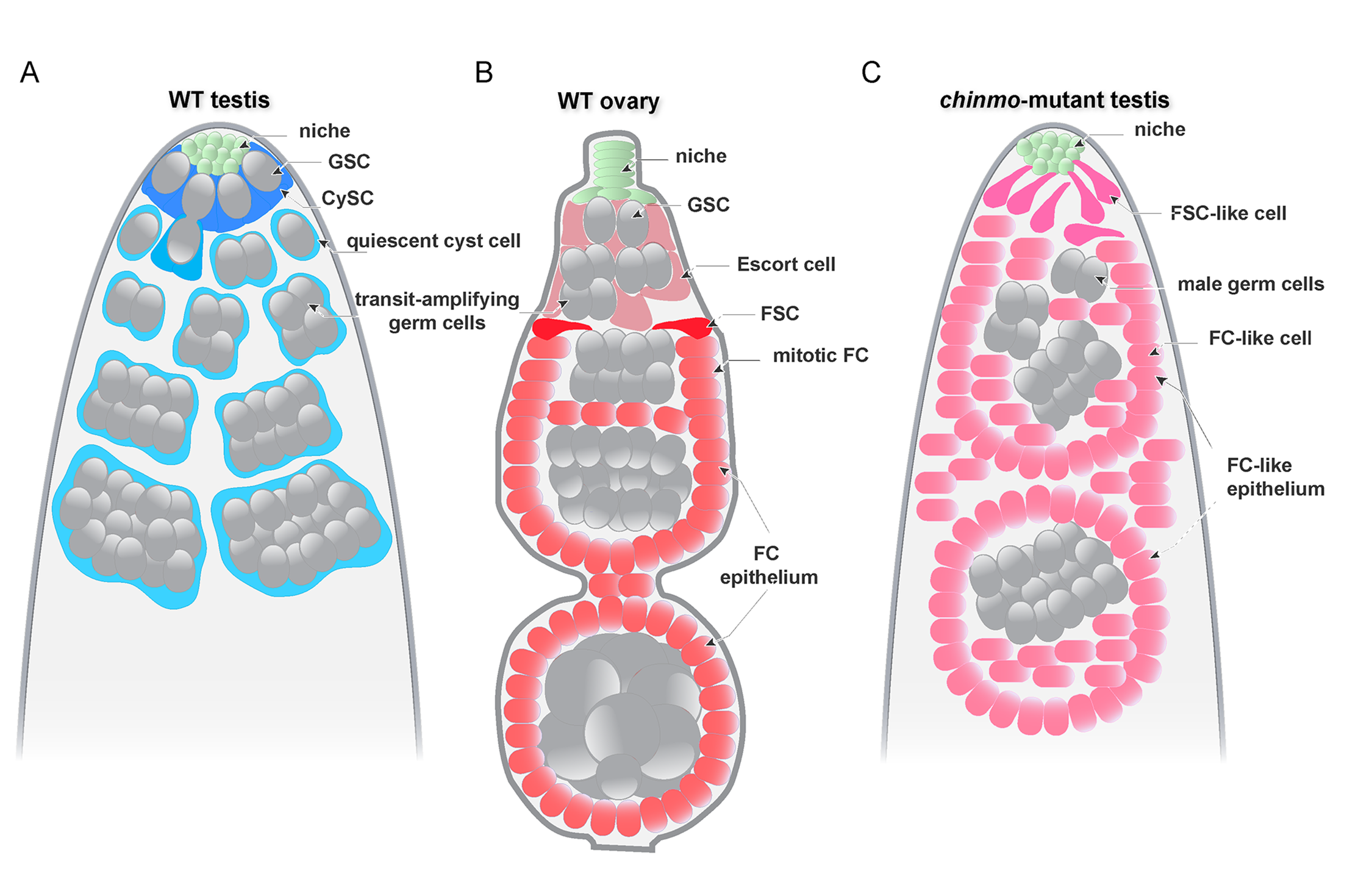

Supplement: S1 Fig — (A) Cyst stem cells (CySCs, dark blue) of a wild-type (WT) testis are mitotic and reside in the same niche (green) as germline stem cells (GSCs, gray). Their daughter cells (cyst cells, light blue) are squamous, quiescent, and encyst the differentiating male germ cells (gray). (B) Follicle stem cells (FSC, dark red) reside in the germaria of a WT ovary. They give rise to follicle cells (FC, light red) that are mitotic and epithelial. FCs form an epithelium that encyst differentiating female germ cells (gray). The ovary also has a niche (green) that supports GSCs (gray). Escort cells (ECs, pink) envelope early female germ cells. (C) In a testis somatically depleted of chinmo, the niche (green) remains and the CySCs transdifferentiate into FSC-like cells (dark pink). These FSC-like cells give rise to FC-like cells (pink) that cannot properly support male germ cells, leading to defective spermatogenesis and infertility. (TIF) [file pbio.3003535.s001.tif]

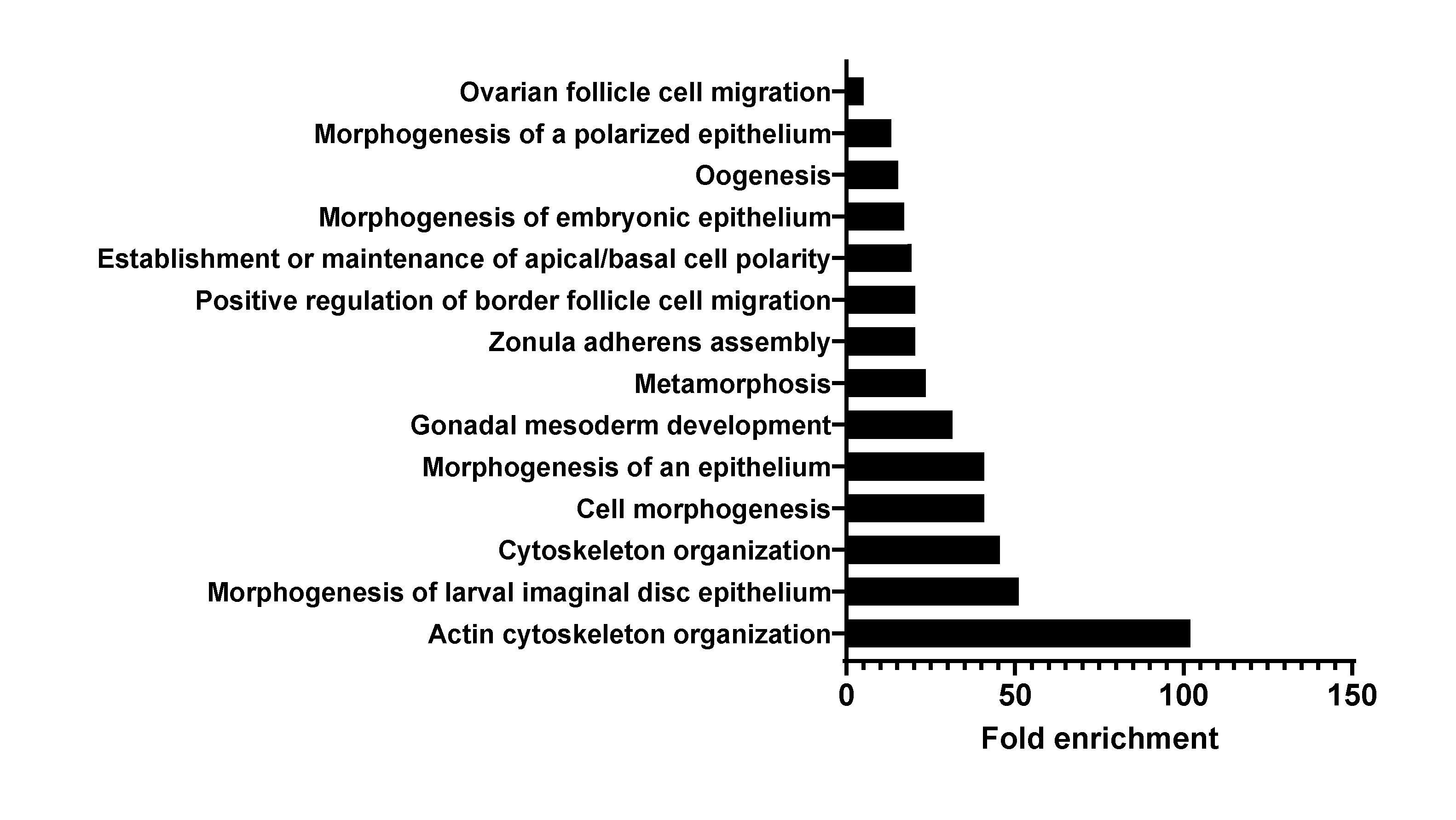

Supplement: S2 Fig — Representative enrichment of upregulated biological processes using Database for Annotation, Visualization and Integrated Discovery (DAVID) classification database. The data underlying the graph shown in the figure can be found in S1 Data. (TIF) [file pbio.3003535.s002.tif]

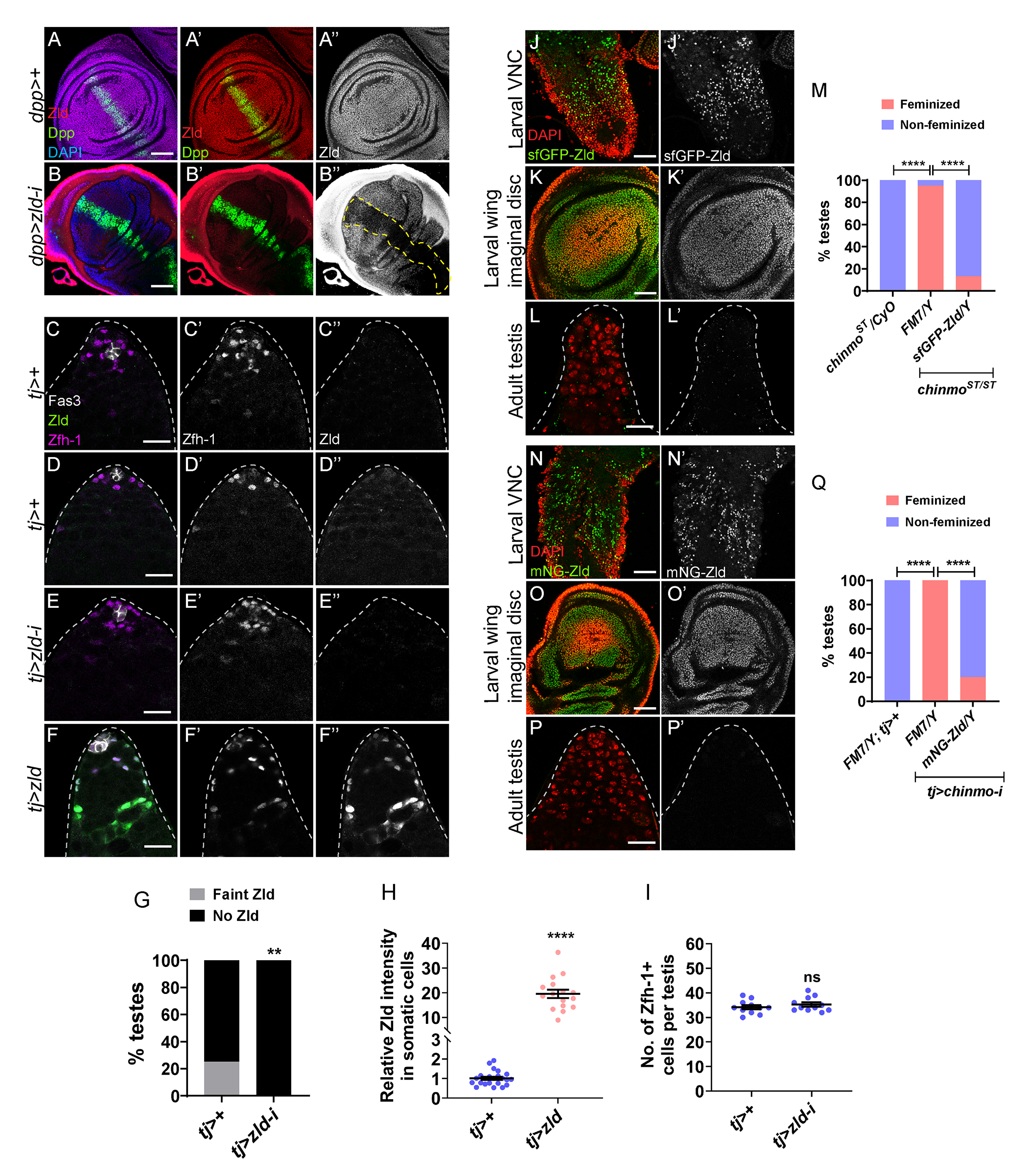

Supplement: S3 Fig — (A, B) Representative confocal images of control (dpp>+) (A) and dpp > zld-i (B) wing imaginal discs stained for Zld (red, grayscale), Dpp domain (green), and DAPI (blue). Yellow dotted lines (B″) show the diminished expression of Zld in dpp > zld-i. (C–F) Representative confocal images from control testes (tj>+) displaying negligible Zld expression (C) or faint Zld expression (D), from tj > zld-i testes (E), and tj > zld testes (F). Testes are stained for Fas3 (grayscale), Zfh-1 (magenta, grayscale), and Zld (green, grayscale). (G) Graph showing the percentage of testes displaying faint (gray) and negligible (black) Zld protein expression in tj>+ (n = 36) and tj > zld-i (n = 25). (H) Graph showing relative Zld protein expression in somatic cells of tj>+ (n = 12) and tj > zld (n = 12) testes. (I) Graph depicting the total number of Zfh-1-positive cells in tj>+ (n = 11) and tj > zld-i (n = 11) testes. (J–L) Representative confocal images of sfGFP-Zld in the larval ventral nerve cord (VNC) (J), wing imaginal disc (K), and adult testis (L). sfGFP-Zld is shown in green and grayscale. DAPI is marked in red. (M) Graph showing the percentage of feminized (pink) and non-feminized (blue) testes in FM7/Y; chinmoST/CyO (n = 15), FM7/Y; chinmoST/ST (n = 20), and sfGFP-Zld/Y; chinmoST/ST (n = 15). (N–P) Representative confocal images of mNG-Zld in larval VNC (N), wing imaginal disc (O), and adult testis (P). mNG-Zld is shown in green and grayscale. DAPI is marked in red. (Q) Graph showing the percentage of feminized (pink) and non-feminized (blue) testes in FM7/Y; tj>+ (n = 15), FM7/Y; tj>chinmo-i (n = 20), and mNG-Zld/Y; tj>chinmo-i (n = 20). Dot plots show individual data points with lines indicating the mean ± SD (H, I). Bar graphs depict the percentage of testes exhibiting the indicated phenotypes (G, M, Q). The data underlying the graphs shown in the figure can be found in S1 Data. Statistical analysis was performed using Student t test (H, I) and Fisher’s exact test (G, M, [file pbio.3003535.s003.tif]

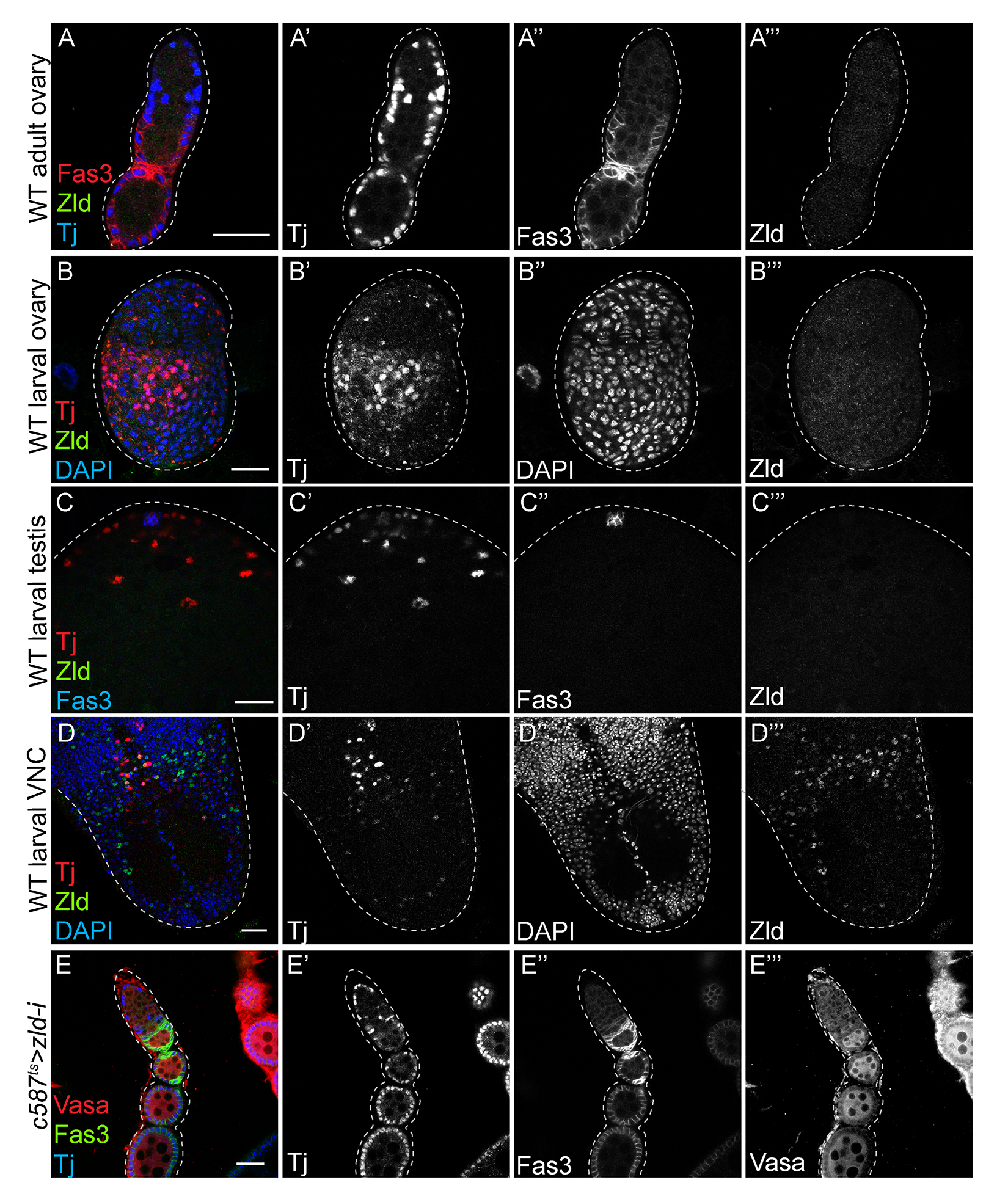

Supplement: S4 Fig — (A) Representative confocal images of a WT adult ovary stained for Tj (blue, grayscale), Fas3 (red, grayscale), and Zld (green, grayscale). (B–D) Representative confocal images of WT larval ovary (B), WT larval testis (C), and WT larval VNC (D) stained for Tj (red, grayscale), Zld (green, grayscale), and Fas3 (blue, grayscale in C). DAPI (blue, grayscale in B, D). (E) Representative confocal images of c587ts>zld-i ovary stained for Tj (blue, grayscale), Fas3 (green, grayscale), and Vasa (red, grayscale). Scale bars: 20 µm. (TIF) [file pbio.3003535.s004.tif]

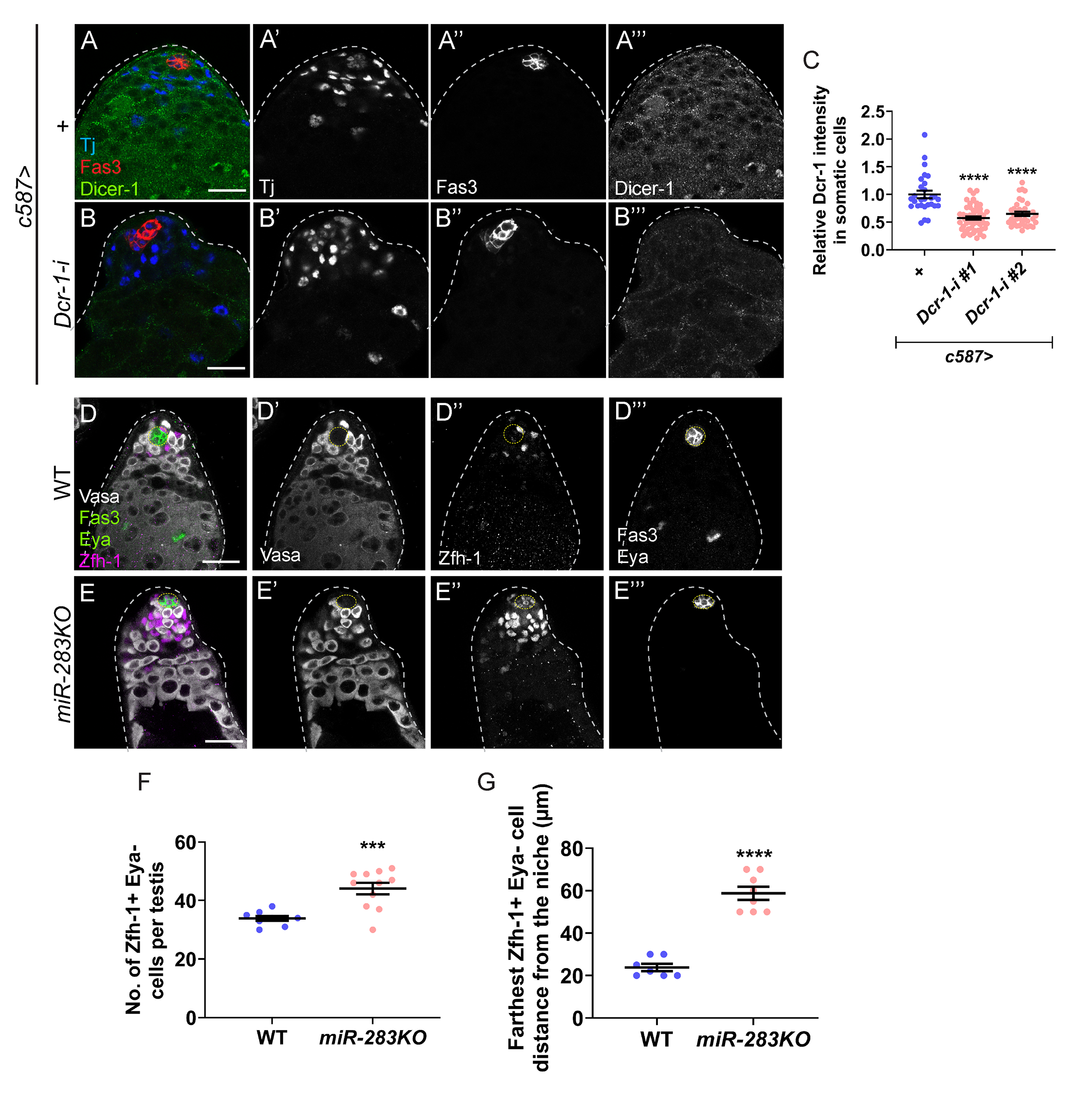

Supplement: S5 Fig — (A, B) Representative confocal images of c587>+ (A) and c587 > Dcr-1-i (B) testes stained for Dcr-1 (green, grayscale), Fas3 (red, grayscale), and Tj (blue, grayscale). (C) Graph showing relative Dcr-1 expression in somatic cells of c587>+ (n = 15), c587 > Dcr-1-i #1 (n = 15), and c587 > Dcr-1-i #2 (n = 9) testes. (D, E) Representative confocal images of WT (D) and miR-283KO (E) testes stained for Vasa (grayscale), Zfh-1 (magenta, grayscale), and Fas3 and Eya (green, grayscale). (F) Graph depicting the total number of Zfh-1-positive, Eya-negative cells in WT (n = 9) and miR-283KO (n = 11) testes. (G) Graph showing distance of the farthest Zfh-1-positive, Eya-negative cells in WT (n = 9) and miR-283KO (n = 11) testes. Dot plots show individual data points with lines indicating the mean ± SD (C, F, G). The data underlying the graphs shown in the figure can be found in S1 Data. Statistical analysis was performed using Student t test (C, F, G) (*** P = 0.0004; **** P < 0.0001). Scale bars: 20 µm. (TIF) [file pbio.3003535.s005.tif]

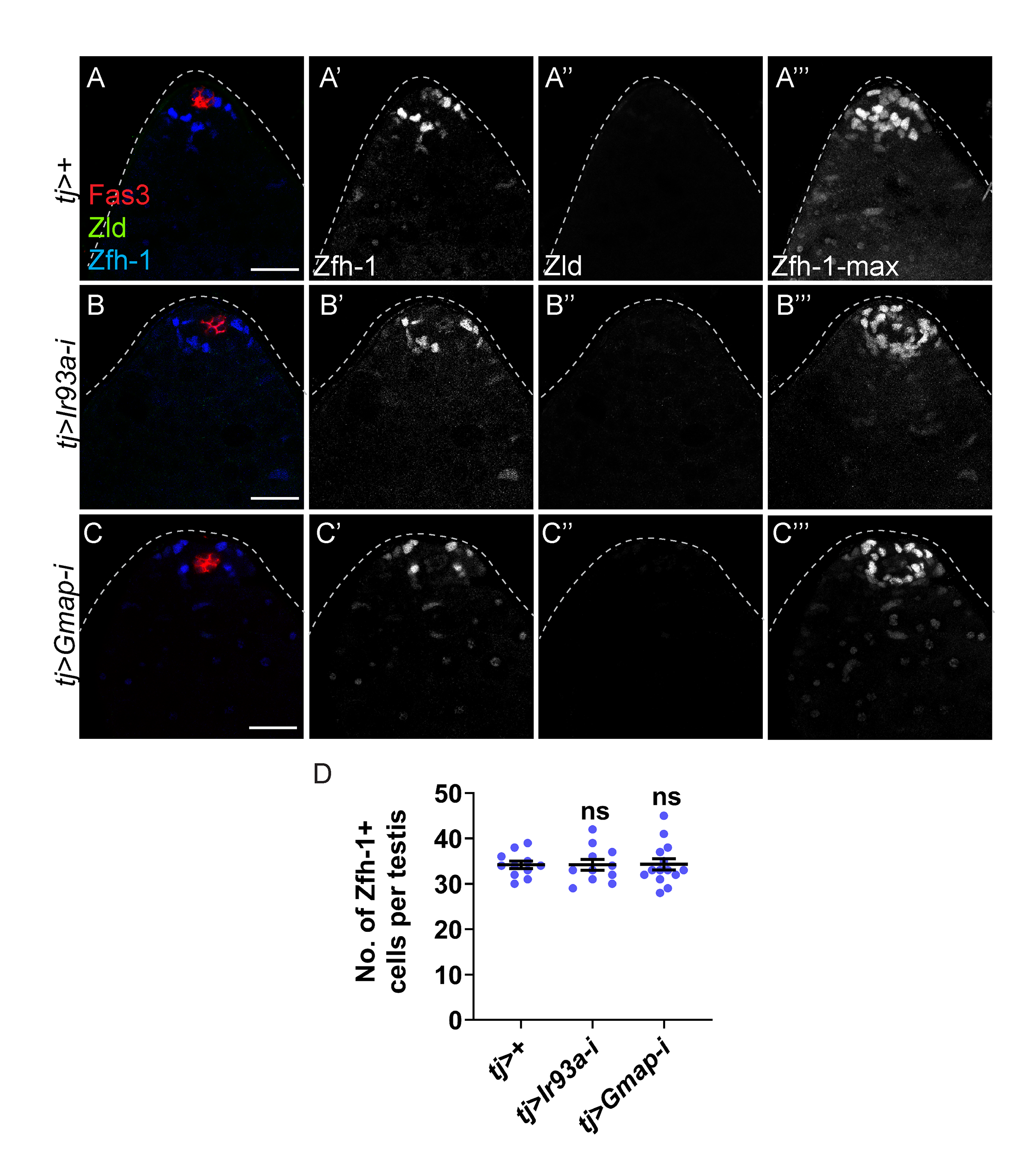

Supplement: S6 Fig — (A–C) Representative confocal images of tj>+ (A), tj > Ir93a-i (B), and tj>Gmap-i (C) testes stained for Zld (green, grayscale), Zfh-1 (blue, grayscale), and Fas3 (red). A″′, B″′, and C″′ show Z-max projections of Zfh-1-expressing cells. (D) Graph depicting the total number of Zfh-1-positive cells in tj>+ (n = 11), tj > Ir93a-i (n = 11), and tj>Gmap-i (n = 12) testes. Dot plots show individual data points with lines indicating the mean ± SD (D). The data underlying the graphs shown in the figure can be found in S1 Data. Statistical analysis was performed using Student t test (D) (ns = not significant). Scale bars: 20 µm. (TIF) [file pbio.3003535.s006.tif]

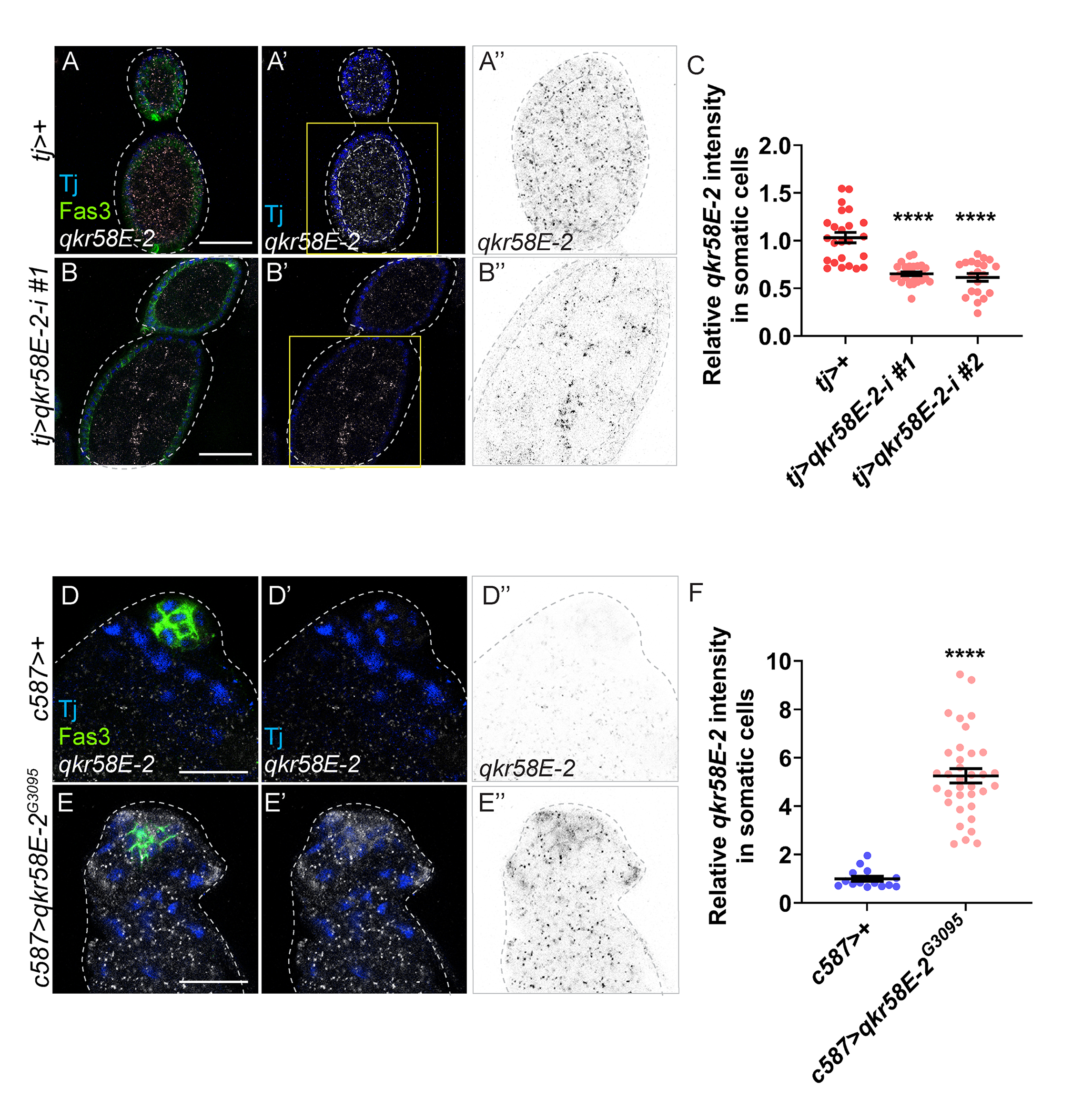

Supplement: S7 Fig — (A, B) Representative confocal images of HCR-FISH for qkr58E-2 mRNA in tj>+ (A) and tj > qkr58E-2-i (B) ovaries. Ovaries are stained for Fas3 (green), Tj (blue), and qkr58E-2 mRNA (grayscale, inverted grayscale). The panels A″ and B″ shows the enlarged view of the inset marked with yellow boxes in A′ and B′. (C) Graph showing relative qkr58E-2 mRNA intensity in somatic cells in tj>+ (n = 8), tj > qkr58E-2-i #1 (n = 10), and tj > qkr58E-2-i #2 (n = 8) ovaries. (D, E) Representative confocal images of HCR-FISH for qkr58E-2 mRNA in c587>+ (D) and c587 > qkr58E-2G3095 (E) testes. The testes are stained for Fas3 (green), Tj (blue), and qkr58E-2 mRNA (grayscale, inverted grayscale). (F) Graph showing relative qkr58E-2 mRNA intensity in somatic cells in c587>+ (n = 8) and c587 > qkr58E-2 G3095 (n = 9) testes. Dot plots show individual data points with lines indicating the mean ± SD (C, F). The data underlying the graphs shown in the figure can be found in S1 Data. Statistical analysis was performed using Student t test (C, F) (**** P < 0.0001). Scale bars: 20 µm. (TIF) [file pbio.3003535.s007.tif]

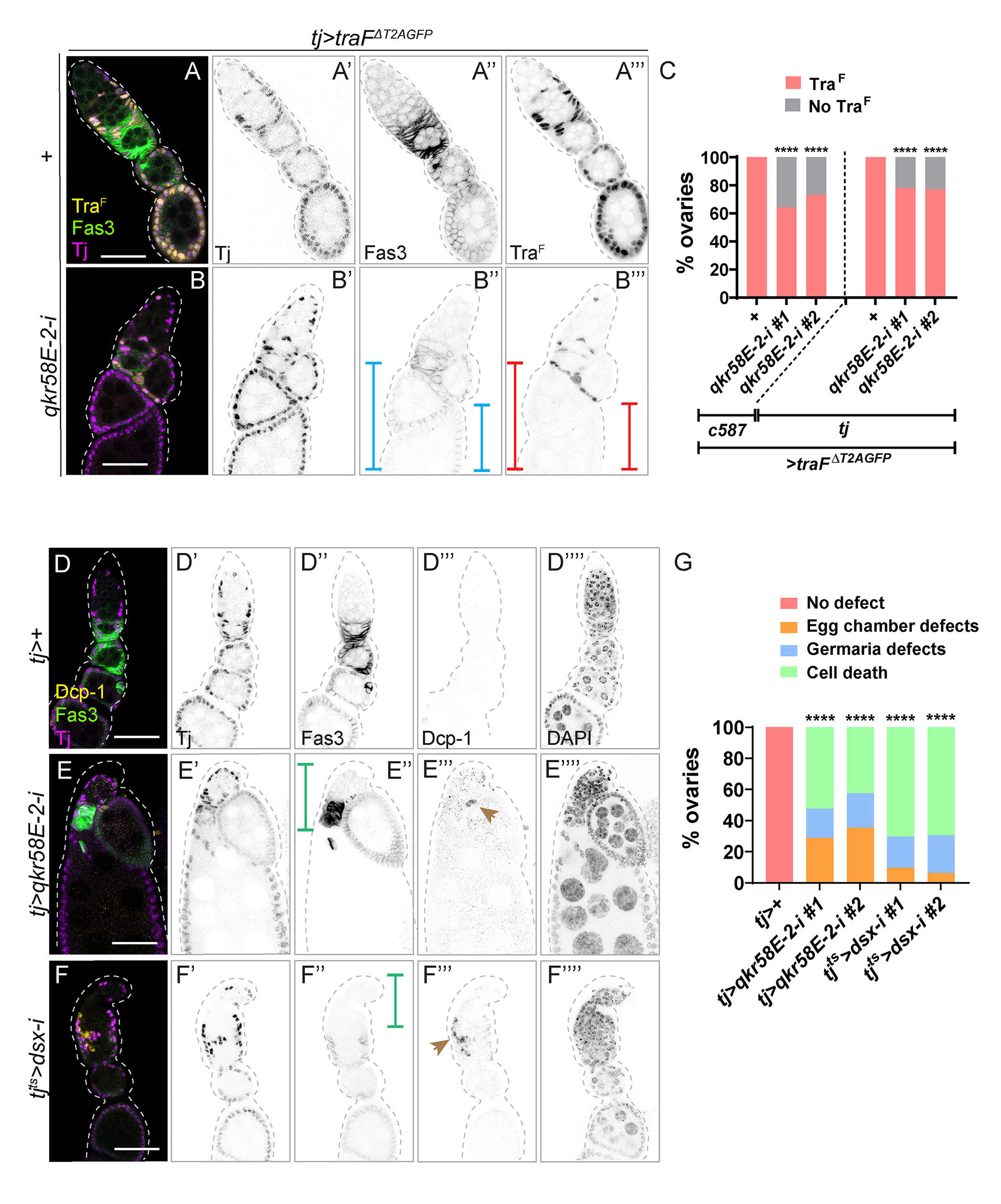

Supplement: S8 Fig — (A, B) Representative confocal images of tj > traFΔT2AGFP + (A) and tj > traFΔT2AGFP; qkr58E-2-i ovaries (B) stained for GFP (indicative of TraF) (yellow, inverted grayscale), Fas3 (green, inverted grayscale), and Tj (magenta, inverted grayscale). Blue and red brackets in B″ and B″′, respectively, show reduced Fas3 and TraF in qkr58E-2-depleted follicle cells. (C) Graph showing the percentage ovaries with (pink) or without (gray) TraF in c587 > traFΔT2AGFP + (n = 65), c587 > traFΔT2AGFP; qkr58E-2-i #1 (n = 98), c587 > traFΔT2AGFP; qkr58E-2-i #2 (n = 71), tj > traFΔT2AGFP + (n = 55), tj > traFΔT2AGFP; qkr58E-2-i #1 (n = 68), and tj > traFΔT2AGFP; qkr58E-2-i #2 (n = 56). (D–F) Representative confocal images of tj>+ (D), tj > qkr58E-2-i (E), and tjts > dsx-i (F) ovaries stained for Dcp-1 (yellow, inverted grayscale), Fas3 (green, inverted grayscale), Tj (magenta, inverted grayscale), and DAPI (inverted grayscale). Green brackets in E″ and F″ show fused egg chambers and abnormal germaria in ovaries somatically depleted for qkr58E-2 (E″) and dsx (F″). Brown arrows in E″′ and F″′ show Dcp-1 expression in follicle cells depleted for qkr58E-2 (E″′) and dsx (F″′). (G) Graph showing defective oogenesis and cell death in tj>+ (n = 72), tj > qkr58E-2-i #1 (n = 52), tj > qkr58E-2-i #2 (n = 37), tjts > dsx-i #1 (n = 58), and tjts > dsx-i #2 (n = 32) ovaries. Bar graphs depict the percentage of ovaries exhibiting the indicated phenotypes (C, G). The data underlying the graphs shown in the figure can be found in S1 Data. Statistical analysis was performed using Fisher’s exact test (C, G) (**** P < 0.0001). Scale bars: 20 µm. (TIF) [file pbio.3003535.s008.tif]

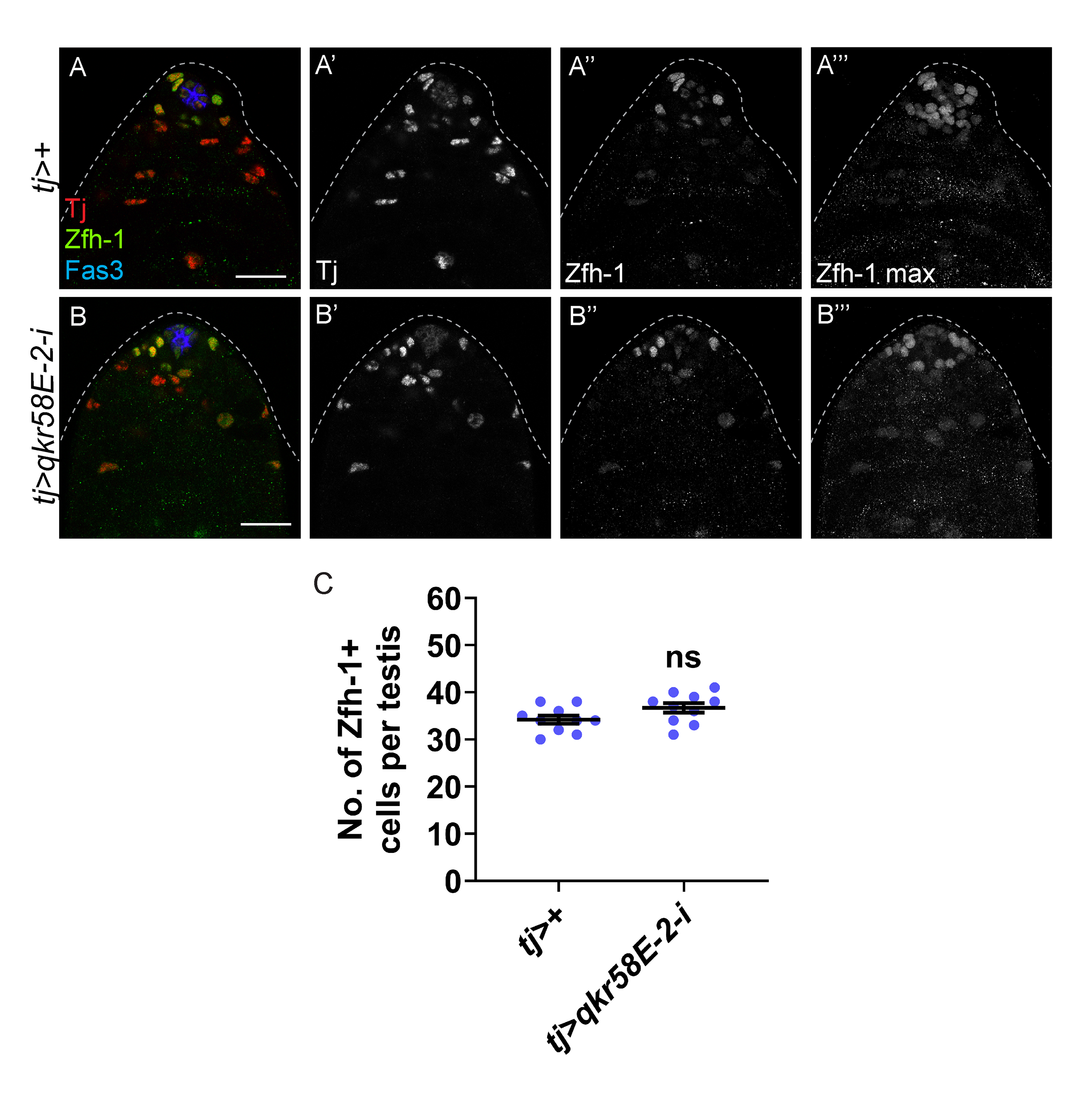

Supplement: S9 Fig — (A, B) Representative confocal images of tj>+ (A) and tj > qkr58E-2-i (B) testes. The testes are stained for Fas3 (blue), Tj (red, grayscale), and Zfh-1 (green, grayscale). A″′ and B″′ show Z-max projections of Zfh-1-expressing cells. (C) Graph depicting the total number of Zfh-1-positive cells in tj>+ (n = 10) and tj > qkr58E-2-i (n = 10) testes. Dot plots show individual data points with lines indicating the mean ± SD (C). The data underlying the graphs shown in the figure can be found in S1 Data. Statistical analysis was performed using Student t test (C) (ns = not significant). Scale bars: 20 µm. (TIF) [file pbio.3003535.s009.tif]

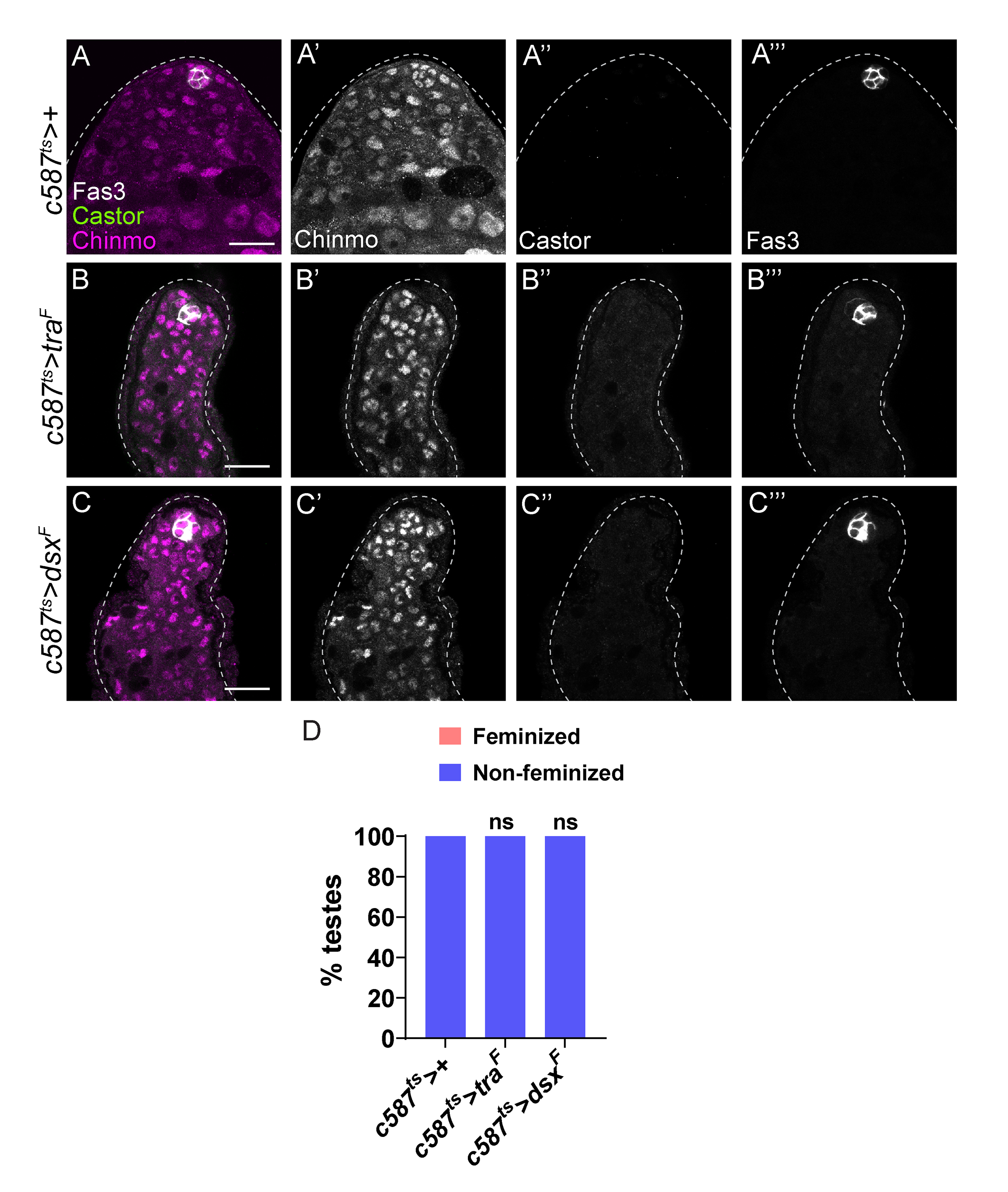

Supplement: S10 Fig — (A–C) Representative confocal images of c587ts>+ (A), c587ts>traF (B), and c587ts>dsxF testes at 20 days of adulthood. The testes are stained for Fas3 (grayscale), Castor (green, grayscale), and Chinmo (magenta, grayscale). (D) Graph showing the percentage of feminized (pink) and non-feminized (blue) testes in c587ts>+ (n = 10), c587ts>traF (n = 10), and c587ts>dsxF (n = 10). Bar graphs depict the percentage of testes exhibiting the indicated phenotypes (D). The data underlying the graphs shown in the figure can be found in S1 Data. Statistical analysis was performed using Fisher’s exact test (D) (ns = not significant). Scale bars: 20 µm. (TIF) [file pbio.3003535.s010.tif]

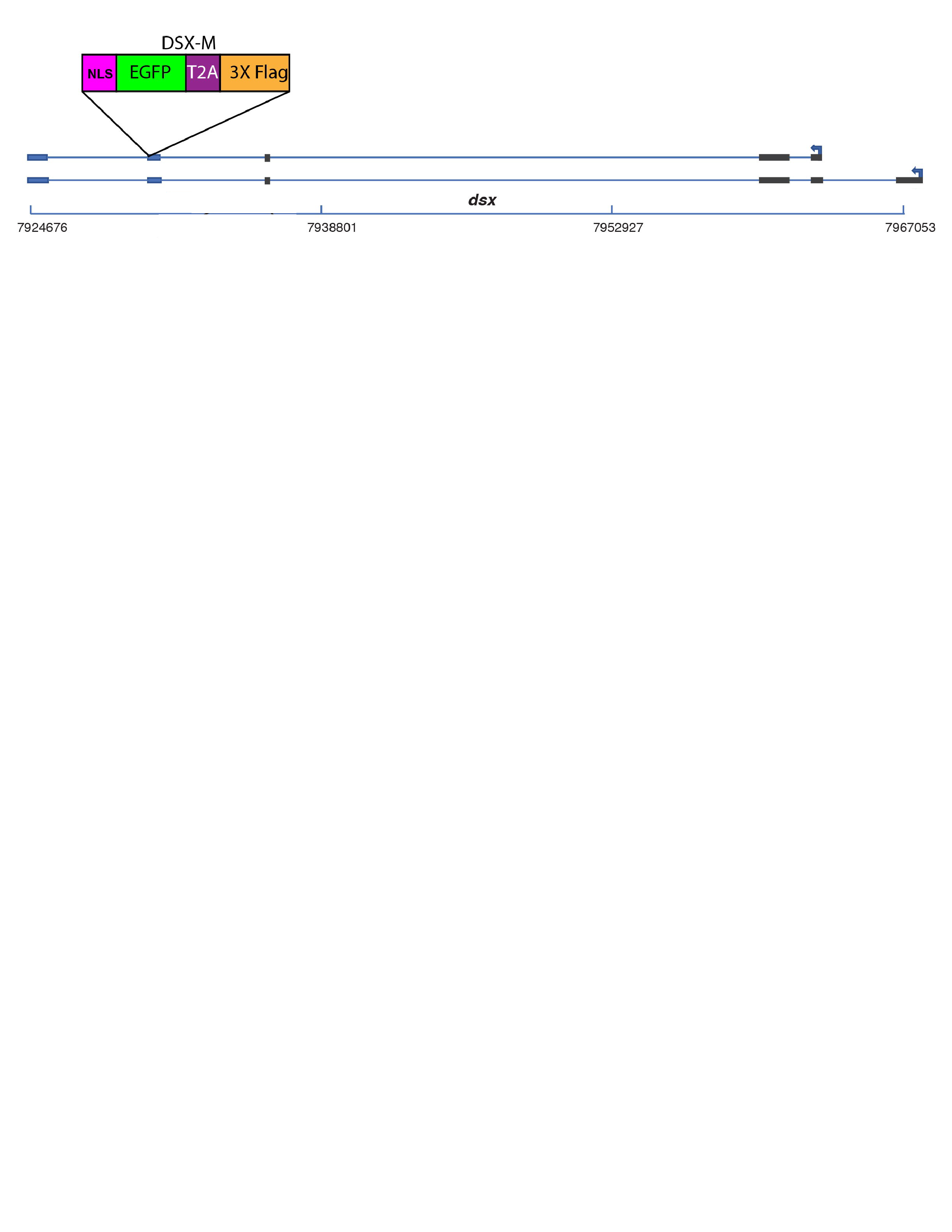

Supplement: S11 Fig — See Materials and methods for detailed information. (TIF) [file pbio.3003535.s011.tif]
