# Supplementary material for: Post-transcriptional suppression of the pioneer factor Zelda protects the adult Drosophila testis from activation of the ovary program
Source: PLoS Biol. 2025 Dec 18;23(12):e3003535. doi: 10.1371/journal.pbio.3003535 (PMC12714197; doi:10.1371/journal.pbio.3003535)
Supplement: S1 Table — All Drosophila stocks are provided with their corresponding RRIDs. (DOCX) [file pbio.3003535.s012.docx]

**S1 Table: List of key materials-antibodies, chemicals, *Drosophila* stocks with RRIDs**

| **Reagent or Resource** | **Source** | **Identifier** | |
| --- | --- | --- | --- |
| **Antibodies** |  | |  |
| Rabbit polyclonal anti-GFP  (1:250) | Invitrogen | | Cat# A6455 |
| Guinea Pig anti-Vasa  (1:500) | Bach Lab | | N/A |
| Rabbit polyclonal anti-Zfh1  (1:200) | K. White (University of Chicago, USA) | | N/A |
| Guinea pig polyclonal anti-Traffic jam (Tj)  (1:1000) | D. Godt (University of Toronto, Canada) | | N/A |
| Chicken polyclonal anti-Vasa  (1:200) | P. Rangan (Icahn School of Medicine at Mount Sinai, USA) | | N/A |
| Rat anti-Chinmo  (1:200) | N. Sokol (Indiana University, USA) | | N/A |
| Guinea Pig anti-Chinmo  (1:250) | N. Sokol | | N/A |
| Chicken polyclonal anti-GFP  (1:250) | Aves Labs | | Cat# GFP-1020 |
| Rabbit anti-cleaved Dcp-1  (1:100) | Cell Signaling | | Cat# 9578  RRID: AB_2721060 |
| Rabbit anti-Zld-N (referred to as anti-Zld)  (1:200) | Rushlow lab | | N/A |
| Rabbit anti-Dicer-1  (1:50) | P. Zamore | | N/A |
| Mouse anti-Fasciclin-3  (1:50) | DSHB | | Cat# 7G10  RRID: AB_528238 |
| Mouse anti-Eyes absent (Eya)  (1:10) | DSHB | | Cat# eya10H6  RRID: AB_528232 |
| Mouse anti-EcR  (1:10) | DSHB | | Cat# Ag10.2 (EcR common)  RRID: AB_10683832 |
| Cy3-AffiniPure Donkey Anti-Mouse IgG  (1:400) | Jackson ImmunoResearch Labs | | Cat# 715-165-150  RRID: AB_2340813 |
| Alexa Fluor 488-AffiniPure Donkey Anti-Rabbit IgG (H+L)  (1:400) | Jackson ImmunoResearch Labs | | Cat# 711-545-152  RRID: AB_2313584 |
| Cy3-AffiniPure Donkey Anti-Rabbit IgG (H+L)  (1:400) | Jackson ImmunoResearch Labs | | Cat# 711-165-152  RRID: AB_2307443 |
| Cy5-AffiniPure Donkey Anti-Rabbit IgG (H+L)  (1:400) | Jackson ImmunoResearch Labs | | Cat# 711-175-152  RRID: AB_2340607 |
| Alexa Fluor 488-AffiniPure Donkey Anti-Rat IgG (H+L)  (1:400) | Jackson ImmunoResearch Labs | | Cat# 712-545-150  RRID: AB_2340683 |
| Cy3-AffiniPure Donkey Anti-Rat IgG (H+L)  (1:400) | Jackson ImmunoResearch Labs | | Cat# 712-165-150,  RRID: AB_2340666 |
| Cy5-AffiniPure Donkey Anti-Rat IgG (H+L)  (1:400) | Jackson ImmunoResearch Labs | | Cat# 712-175-150,  RRID: AB_2340671 |
| Alexa Fluor 488 AffiniPure Donkey Anti-Chicken IgY (IgG) (H+L)  (1:400) | Jackson ImmunoResearch Labs | | Cat# 703-545-155,  RRID: AB_2340375 |
| Cy3-AffiniPure Donkey Anti-Chicken IgY (IgG) (H+L)  (1:400) | Jackson ImmunoResearch Labs | | Cat# 703-165-155,  RRID: AB_2340363 |
| Cy5-AffiniPure Donkey Anti-Chicken IgY (IgG) (H+L)  (1:400) | Jackson ImmunoResearch Labs | | Cat# 703-175-155,  RRID: AB_2340365 |
| Cy3-AffiniPure Donkey Anti-Guinea Pig IgG  (1:400) | Jackson ImmunoResearch Labs | | Cat# 706-165-148,  RRID: AB_2340460 |
| Cy5-AffiniPure Donkey Anti-Guinea Pig IgG (H+L)  (1:400) | Jackson ImmunoResearch Labs | | Cat# 706-175-148,  RRID: AB_2340462 |
| **Chemicals, peptides, and recombinant proteins** |  | |  |
| VECTASHIELD Mounting Medium with DAPI | Vector Laboratories | | Cat# H-1200,  RRID: AB_2336790 |
| VECTASHIELD Mounting | Vector Laboratories | | Cat# H-1000,  RRID: AB_2336789 |
| Paraformaldehyde, 16% w/v aq. soln., methanol free (PFA) | Thermo Fisher Scientific | | Cat# 43368-9L |
| Heparin | Sigma-Aldrich | | Cat# H4784 |
| tRNA | Roche | | Cat# 10109495001 |
| Protector RNase Inhibitor | Roche | | Cat# 3335399001 |
| 20 x Saline Sodium Citrate (SSC) | Thermo Fisher | | Cat# 15557-044 |
| Proteinase K | Thermo Fisher Scientific | | Cat# EO0491 |
| Glycine | Thermo Fisher Scientific | | Cat# BP381-500 |
| Diethyl pyrocarbonate (DEPC) | MilliporeSigma | | Cat# D5758 |
| Molasses | Labscientific | | Cat# FLY-8008-16 |
| Agar | Mooragar | | Cat# 41004 |
| Cornmeal | LabScientific | | Cat# FLY-8010-20 |
| Yeast | LabScientific | | Cat# FLY-8040-20F |
| Tegosept | Sigma | | Cat# H3647-1KG |
| Reagent alcohol | Fisher | | Cat# A962P4 |
| Propionic acid | Fisher | | Cat# A258500 |
| In Situ HCR v3.0 mRNA Imaging Kit (HCR probe sets, HCR amplifiers, HCR buffers (Probe hybridization buffer, Probe wash buffer, Amplification buffer) | Molecular Instruments | | Custom |
| ***Drosophila* stocks with RRIDs** |  | |  |
| *D. melanogaster*; Oregon-R | Bach lab | | N/A |
| *D. melanogaster*; chinmo^ST^/(CyO) | E. Matunis (The Johns Hopkins University School of Medicine, USA) | | N/A |
| *D. melanogaster; P[GawB]NP1624/CyO (tj-GAL4)* | Bach lab | | Kyoto Stock Center: 104055 Flybase: [FBst0302922](http://flybase.org/reports/FBst0302922) |
| *D. melanogaster*; *P[GawB]C587, w**  (*c587-GAL4*) | R. Lehmann (Whitehead Institute, MIT, USA) | | BDSC_ 67747  Flybase: RRID:BDSC_67747 |
| *D. melanogaster; y^1^ v^1^; P[TRiP.HMS00036]attP2/TM3, Sb (chinmo-RNAi)* | BDSC | | BDSC_33638  FlyBase: [FBst0033638](http://flybase.org/reports/FBst0033638) |
| *D. melanogaster* ; *y^1^ sc* v^1^ sev^21^; P[TRiP.HMC05346]attP40*  *(chinmo-RNAi)* | BDSC | | BDSC_62873  FlyBase: [FBst0062873](http://flybase.org/reports/FBst0062873) |
| *D. melanogaster; w^1118^; P[w[+mC]=UAS-lacZ.NZ]J312 on III* | BDSC | | BDSC_3956 FlyBase: [FBst0003956](http://flybase.org/reports/FBst0003956) |
| *D. melanogaster; w^1118^; P[w[+mC]=UAS-lacZ.NZ]20b.* | Kyoto Stock Center | | Kyoto_107531  FlyBase: [FBst0306182](http://flybase.org/reports/FBst0306182) |
| *D. melanogaster*; *w*; P[w[+mC]=tubP-GAL80^ts^]2/TM2* | Bach lab | | BDSC_7017 FlyBase: [FBst0007017](http://flybase.org/reports/FBst0007017) |
| *D. melanogaster*; *w*; P[w[+mC]=tubP-GAL80^ts^]20; TM2/TM6B, Tb^1^* | Bach lab | | BDSC_7019  FlyBase: [FBst0007019](http://flybase.org/reports/FBst0007019) |
| *D. melanogaster*; *UAS-zld shmIR (zygotic) 27F on III,* referred to as *zld-RNAi* | Rushlow lab | | N/A |
| *D. melanogaster*; *mNeonGreen-Zld* | M. Mir (University of Pennsylvania, The Perelman School of Medicine, USA) | | N/A |
| *D. melanogaster*; *sfGFP-zld* | M. Harrison (University of Wisconsin-Madison, USA) | | N/A |
| *D. melanogaster; act>stop>lacZ/Bl; dpp-gal4, UAS-GFP/TM6B (dpp>GFP)* | Bach Lab | | N/A |
| *D. melanogaster; w*;mir-1011[KO] Ir93a[mir-1011-KO]/TM3, Sb^1^ Ser^1^* | BDSC | | BDSC_58887  FlyBase: FBst0058887 |
| *D. melanogaster; w* mir-283KO* | BDSC | | BDSC_58912  FlyBase: FBst0058912 |
| *D. melanogaster; w*; P[UAS-mCherry.mirbft.sponge.V2]attP40/*  *CyO; P[UAS-mCherry.mir bft.sponge.V2]attP2*  *Used to sequester miR-263a (which is another name for bft)* | BDSC | | BDSC_61402  FlyBase: FBst0061402 |
| *D. melanogaster; w[*]; P[UAS-mCherry.mir-283.sponge.V2]attP40; P[UAS-mCherry.mir-283.sponge.V2]attP2* | BDSC | | BDSC_61415  FlyBase: [FBst0061415](https://flybase.org/reports/FBst0061415) |
| *D. melanogaster; w[*]; P[UAS-mCherry.mir-1011.sponge.V2]attP40; P[UAS-mCherry.mir-1011.sponge.V2]attP2* | BDSC | | BDSC_ 61494  FlyBase: [FBst0061494](https://flybase.org/reports/FBst0061494) |
| *D. melanogaster; w*; P[UAS-DsRed-mir-263a]/CyO* | BDSC | | BDSC_59893  FlyBase: FBst0059893 |
| *D. melanogaster; w*; P[UAS-DsRed-mir-263a]* | BDSC | | BDSC_59894  FlyBase: FBst0059894 |
| *D. melanogaster; w*; P[UAS-mir-1011.S]attP2* | BDSC | | BDSC_60665  FlyBase: FBst0060665 |
| *D. melanogaster; w*; P[UAS-mir-1011.S]attP16* | BDSC | | BDSC_60666  FlyBase: FBst0060666 |
| *D. melanogaster; w*; P[UAS-mCherry.scramble.sponge]attP40;*  *P [UAS-mCherry.scramble.sponge]attP2* | BDSC | | BDSC_61501  FlyBase: [FBst0061501](http://flybase.org/reports/FBst0061501) |
| *D. melanogaster*; *UAS-traF^ΔT2AGFP^* | Bach Lab | | N/A |
| *D. melanogaster*; *UAST-zld #2M on II,* referred to as *UAS-zld* | Rushlow lab | | N/A |
| *D. melanogaster*; *y^1^ sc* v^1^ sev^21^;P[TRiP.HMC03966]*  *attP40/CyO (qkr58E-2-RNAi) #1* | BDSC | | BDSC_55279  FlyBase: [FBst0055279](http://flybase.org/reports/FBst0055279) |
| *D. melanogaster*; *y^1^ sc* v^1^ sev^21^; P[TRiP.HMS05750]attP40 (qkr58E-2-RNAi) #2* | BDSC | | BDSC_67912  FlyBase: [FBst0067912](http://flybase.org/reports/FBst0067912) |
| *D. melanogaster*; *P[EP]qkr58E-2^G3095^* | Kyoto Stock Center | | Kyoto_10922  FlyBase: [FBst1022922](http://flybase.org/reports/FBst1022922) |
| *D. melanogaster; y^1^ sc* v^1^ sev^21^; P[TRiP.GLV21010]attP2 (dsx-RNAi) #1* | BDSC | | BDSC_35645  FlyBase: [FBst0035645](http://flybase.org/reports/FBst0035645) |
| *D. melanogaster; y^1^ sc* v^1^ sev^21^; P[TRiP.HMC03795]attP40 (dsx-RNAi) #2* | BDSC | | BDSC_55646  FlyBase: [FBst0055646](http://flybase.org/reports/FBst0055646) |
| *D. melanogaster; w^1118^; P[GD11429]v24667 (Dcr-1-RNAi)#1* | VDRC | | VDRC_24667  FlyBase: [FBst0455535](https://flybase.org/reports/FBst0455535) |
| *D. melanogaster; y^1^ sc*v1 sev21; P[TRiP.HMS02594]attP40 (Dcr-1-RNAi)#2* | BDSC | | BDSC_42901  FlyBase: [FBst0042901](https://flybase.org/reports/FBst0042901) |
| *D. melanogaster; y^1^ v^1^; P[TRiP.HMJ30268]attP40 (Ir93a-RNAi)* | BDSC | | BDSC_63700  FlyBase: FBst0063700 |
| *D. melanogaster; y^1^ sc* v^1^ sev^21^; P[TRiP.HMC05736]attP40 (Gmap-RNAi)* | BDSC | | BDSC_64863  FlyBase: [FBst0064863](http://flybase.org/reports/FBst0064863) |
| *D. melanogaster; w^1118^; P[w+mC=UAS-Dcr-2.D]10 (UAS-Dcr-2)* | BDSC | | BDSC_24651  FlyBase: [FBst0024651](http://flybase.org/reports/FBst0024651) |
| *D. melanogaster; y^1^ v^1^; P[TRiP.HMC03114]attP2/TM3, Sb*  *(EcR-RNAi)* | BDSC | | BDSC_50712  FlyBase: [FBst0050712](https://flybase.org/reports/FBst0050712) |
| *D. melanogaster; w^1118^; P[w[+mC]=UAS-tai.B]JB1* | BDSC | | BDSC_6378  FlyBase: [FBst0006378](https://flybase.org/reports/FBst0006378) |
| *D. melanogaster; w^1118^; P[w[+mC]=UAS-EcR.C]TP1-4* | BDSC | | BDSC_6868  FlyBase: [FBst0006868](https://flybase.org/reports/FBst0006868) |
| *D. melanogaster; y^1^ w*; P[w[+mC]=UAS-dsx.F]24-3*  *(UAS-dsx^F^)* | BDSC | | BDSC_44223  FlyBase: [FBst0044223](http://flybase.org/reports/FBst0044223) |
| *D. melanogaster; w^1118^; P[w[+mC]=UAS-tra.F]20J7*  *(UAS-tra^F^)* | BDSC | | BDSC_4590  FlyBase: [FBst0004590](http://flybase.org/reports/FBst0004590) |
| *D. melanogaster; Dsx^M^::GFP* | B. Oliver lab (NIH/NIDDK, USA) | | N/A |
| *D. melanogaster*: *w^1118^; PBac [+mDint2]=vas-Cas9]VK00037/CyO, P[w[+mC]=Tb[1]]Cpr[CyO-A]* | BDSC | | BDSC_56552 |
| *D. melanogaster; FlyFos019934(pRedFlp-Hgr)(Yp1[35321]::2XTY1-SGFP-V5-preTEV-BLRP-3XFLAG)dFRT* (*Yp1::GFP*) | VDRC | | VDRC_318746  Flybase: FBal0339460 |
