# Supplementary material for: Post-transcriptional suppression of the pioneer factor Zelda protects the adult Drosophila testis from activation of the ovary program
Source: PLoS Biol. 2025 Dec 18;23(12):e3003535. doi: 10.1371/journal.pbio.3003535 (PMC12714197; doi:10.1371/journal.pbio.3003535)
Supplement: S2 Table — (DOCX) [file pbio.3003535.s013.docx]

| **S2 Table: Genotypes analyzed in main body and supplementary data** | | |
| --- | --- | --- |
| **Figures** | **Full genotype** | **Abbreviation used in Figure** |
| 1A | *c587-Gal4/Y; tub-Gal80^ts^/+; UAS-lacZ/+* | *c587^ts^>lacZ* |
| 1B | *c587-Gal4/Y; tub-Gal80^ts^/+; +/UAS-chinmo-RNAi* | *c587^ts^>chinmo-i* |
| 1C | *Oregon-R* | WT |
| 1D | *w/Y; chinmo^ST^/chinmo^ST^* | *chinmo^ST/ST^* |
| 1E | *c587-Gal4/Y; tub-Gal80^ts^/+; UAS-lacZ/+* | *c587^ts^>lacZ* |
| 1E | *c587-Gal4/Y; tub-Gal80^ts^/+; +/UAS-chinmo-RNAi* | *c587^ts^>chinmo-i* |
| 1F | *Oregon-R* | WT |
| 1F | *w/Y; chinmo^ST^/chinmo^ST^* | *chinmo^ST/ST^* |
| 1G | *w/Y; tj-Gal4/UAS-lacZ; UAS-Dcr-2/UAS-lacZ* | *tj>lacZ; lacZ* |
| 1H | *w/Y; tj-Gal4/UAS-chinmo-RNAi; UAS-Dcr-2/UAS-lacZ* | *tj>chinmo-i; lacZ* |
| 1I | *w/Y; tj-Gal4/UAS-chinmo-RNAi; UAS-Dcr-2/UAS-zld-RNAi* | *tj>chinmo-i; zld-i* |
| 1J (left side) | *w/Y; tj-Gal4/UAS-lacZ; UAS-Dcr-2/UAS-lacZ* | *tj>lacZ; lacZ* |
| 1J (left side) | *w/Y; tj-Gal4/UAS-chinmo-RNAi; UAS-Dcr-2/UAS-lacZ* | *tj>chinmo-i; lacZ* |
| 1J (left side) | *w/Y; tj-Gal4/UAS-chinmo-RNAi; UAS-Dcr-2/UAS-zld-RNAi* | *tj>chinmo-i; zld-i* |
| 1J (right side) | *c587-Gal4/Y; chinmo^ST^/CyO* | *c587>chinmo^ST^/CyO* |
| 1J (right side) | *c587-Gal4/Y; chinmo^ST^/chinmo^ST^* | *c587>chinmo^ST/ST^* |
| 1J (right side) | *c587-Gal4/Y; chinmo^ST^/chinmo^ST^; UAS-zld-RNAi/+* | *c587>chinmo^ST/ST^; zld-i* |
|  |  |  |
| 2A | *Oregon-R* | WT |
| 2B | *w/Y; chinmo^ST^/chinmo^ST^* | *chinmo^ST/ST^* |
| 2C | *Oregon-R* | WT |
| 2C | *w/Y; chinmo^ST^/chinmo^ST^* | *chinmo^ST/ST^* |
| 2D | *c587-Gal4/Y; +/+* | *c587>+* |
| 2E | *c587-Gal4/Y; +/UAS-Dcr-1-RNAi* | *c587>Dcr-1-i* |
| 2F | *c587-Gal4/Y; +/+* | *c587>+* |
| 2F | *c587-Gal4/Y; +/UAS-Dcr-1-RNAi* | *c587>Dcr-1-i#1* |
| 2F | *c587-Gal4/Y; +/UAS-Dcr-1-RNAi* | *c587>Dcr-1-i#2* |
| 2F | *c587-Gal4/Y; tub-Gal80^ts^/UAS-zld; +/+* | *c587^ts^>zld* |
| 2G | *c587-Gal4/Y; +/+* | *c587>+* |
| 2G | *c587-Gal4/Y; +/UAS-Dcr-1-RNAi* | *c587>Dcr-1-i#1* |
| 2G | *c587-Gal4/Y; +/UAS-Dcr-1-RNAi* | *c587>Dcr-1-i#2* |
|  |  |  |
| 3B, C, D, K, L, M | *Oregon-R* | WT |
| 3E, F, G, K, L, M | *c587-Gal4/Y; tub-Gal80^ts^/+; UAS-chinmo-RNAi/+* | *c587^ts^>chinmo-i* |
| 3H, K | *w/Y; +/+; miR-1011KO/miR-1011KO* | miR mutant or miR Sponge |
| 3I, L | *w/Y; tj-Gal4/UAS-miR-263aSP; +/UAS-miR-263a-SP* | miR mutant or miR Sponge |
| 3J, M | *w, miR-283KO/Y* | miR mutant or miR Sponge |
| 3N, R | *Oregon-R* | WT |
| 3O, R | *w/Y; +/+; miR-1011KO/miR-1011KO* | *miR-1011KO* |
| 3P, R | *c587-Gal4/Y; +/UAS-miR-263aSP; +/UAS-miR-263aSP* | *c587>miR-263aSP* |
| 3Q, R | *w, miR-283KO/Y* | *miR-283KO* |
|  |  |  |
| 4A, L, M | *Oregon-R* | WT |
| 4B, L, M | *w/Y; +/+; miR-1011KO/miR-1011KO* | *miR-1011KO* |
| 4C, L, M | *w, miR-283KO/Y* | *miR-283KO* |
| 4D, L, M | *c587-Gal4/Y; +/UAS-ScrambleSP; +/UAS-ScrambleSP* | *c587>Scramble-SP* |
| 4E, L, M | *c587-Gal4/Y; +/UAS-miR-263aSP; +/UAS-miR-263aSP* | *c587>miR-263aSP* |
| 4F, L, M | *c587-Gal4/Y; +/UAS-miR-283SP; +/UAS-miR-283SP* | *c587>miR-283SP* |
| 4G, L, M | *c587-Gal4/Y; +/UAS-miR-1011SP; +/UAS-miR-1011SP* | *c587>miR-1011SP* |
| 4H, L, M | *c587-Gal4/Y; +/UAS-miR-263aSP; +/UAS-miR-1011SP* | *c587>miR-263aSP;*  *miR-1011SP* |
| 4I, L, M | *c587-Gal4/Y; +/UAS-miR-263aSP; +/UAS-miR-283SP* | *c587>miR-263aSP;*  *miR-283SP* |
| 4J, L, M | *c587-Gal4/Y; +/UAS-miR-283SP; +/UAS-miR-1011SP* | *c587>miR-283SP;*  *miR-1011SP* |
| 4K, L, M | *c587-Gal4/Y; UAS-miR-283SP/UAS-miR-263aSP; +/UAS-miR-1011SP* | *c587>miR-283SP,miR-263aSP, miR-1011SP* |
|  |  |  |
| 5A, D | *c587-Gal4/Y; tub-Gal80^ts^/+; +/+* | *c587^ts^>+* |
| 5B, D | *c587-Gal4/Y; tub-Gal80^ts^/+; UAS-chinmo-RNAi/+* | *c587^ts^>chinmo-i* |
| 5C, D | *c587-Gal4/Y; tub-Gal80^ts^/UAS-miR-263a; UAS-miR-1011/UAS-chinmo-RNAi* | *c587^ts^>miR-263a; miR-1011; chinmo-i* |
| 5D | *c587-Gal4/Y; tub-Gal80^ts^/UAS-miR-263a; UAS-chinmo-RNAi/+* | *c587^ts^>miR-263a; chinmo-i* |
| 5D | *c587-Gal4/Y; tub-Gal80^ts^/UAS-miR-1011; UAS-chinmo-RNAi/+* | *c587^ts^>miR-1011; chinmo-i* |
|  |  |  |
| 6B, C, F, G, I, J | *c587-Gal4/Y; tub-Gal80^ts^/UAS-zld; UAS-traF*^Δ^*^T2AGFP^/+* | *c587^ts^>traF*^Δ^*^T2AGFP^; zld* |
| 6D, E | *c587-Gal4/Y; tub-Gal80^ts^/+; UAS-traF*^Δ^*^T2AGFP^/UAS-chinmo-RNAi* | *c587^ts^>traF*^Δ^*^T2AGFP^; chinmo-i* |
| 6H | *c587-Gal4/Y; tub-Gal80^ts^/UAS-zld; +/+* | *c587^ts^>zld* |
|  |  |  |
| 7A-C | *c587-Gal4/Y; tub-Gal80^ts^/+; UAS-chinmo-RNAi/+* | *c587^ts^>chinmo-i* |
| 7D, H | *Oregon-R* | WT testis |
| 7E, H | *Oregon-R* | WT ovary |
| 7F-H | *c587-Gal4/Y; tub-Gal80^ts^/UAS-zld; +/+* | *c587^ts^>zld* |
| 7I, J, L | *c587-Gal4/Y; tub-Gal80^ts^/UAS-zld; UAS-traF*^Δ^*^T2AGFP^/+* | *c587^ts^>traF*^Δ^*^T2AGFP^; zld* |
| 7K, L | *c587-Gal4/Y; UAS-qkr58E-2-RNAi/UAS-zld; tubGal80^ts^/UAS-traF*^Δ^*^T2AGFP^* | *c587^ts^>traF*^Δ^*^T2AGFP^; zld; qkr58E-2-i #1* |
| 7M | *c587-Gal4/Y; tub-Gal80^ts^/+; +/+* | *c587^ts^>+* |
| 7M | *c587-Gal4/Y; tub-Gal80^ts^/+; UAS-chinmo-RNAi/+* | *c587^ts^>chinmo-i* |
| 7M | *c587-Gal4/Y; tub-Gal80^ts^/UAS-qkr58E-2-RNAi#1; UAS-chinmo-RNAi/+* | *c587^ts^>qkr58E-2-i #1; chinmo-i* |
| 7M | *c587-Gal4/Y; tub-Gal80^ts^/UAS-qkr58E-2-RNAi#2; UAS-chinmo-RNAi/+* | *c587^ts^>qkr58E-2-i #2; chinmo-i* |
|  |  |  |
| 8A, D | *c587-Gal4/Y; +/+; UAS-traF*^Δ^*^T2AGFP^/+* | *c587>traF*^Δ^*^T2AGFP^* |
| 8B-D | *c587-Gal4/Y; +/UAS-qkr58E-2^G3095^; UAS-traF*^Δ^*^T2AGFP^/+* | *c587>traF*^Δ^*^T2AGFP^; qkr58E-2* |
| 8E | *c587-Gal4/Y; +/+* | *c587>+* |
| 8E | *c587-Gal4/Y; +/UAS-qkr58E-2^G3095^* | *c587>qkr58E-2* |
|  |  |  |
| 9A-D | *c587-Gal4/Y; tub-Gal80^ts^/UAS-zld; +/+* | *c587^ts^>zld* |
|  |  |  |
| 10A, E | *c587-Gal4/w; tub-Gal80^ts^/+* | *c587^ts^>+* ovary |
| 10B, E | *c587-Gal4/w; tub-Gal80^ts^; UAS-EcR-i/+* | *c587^ts^>EcR-i* ovary |
| 10C, E | *c587-Gal4/Y; tub-Gal80^ts^/+* | *c587^ts^>+* testis |
| 10D, E | *c587-Gal4/Y; tub-Gal80^ts^/+; UAS-chinmo-RNAi/+* | *c587^ts^>chinmo-i* testis |
| 10F, G, H | *c587-Gal4/Y; tub-Gal80^ts^/UAS-zld; +/+* | *c587^ts^>zld* |
|  |  |  |
| 11A, F, G | *c587-Gal4/w; tub-Gal80^ts^/+* | *c587^ts^>+* |
| 11B, F, G | *c587-Gal4/Y; tub-Gal80^ts^/UAS-zld; +/+* | *c587^ts^>zld* |
| 11C, F, G | *c587-Gal4/Y; tub-Gal80^ts^/UAS-zld; UAS-EcR-i/+* | *c587^ts^>zld; EcR-i* |
| 11D, F, G | *w/Y; tj-Gal4/+; +/+* | *tj>+* |
| 11E, F, G | *w/Y; tj-Gal4/+; UAS-EcR, UAS-tai* | *tj>EcR, tai* |
|  |  |  |
| 12A, B | *Dsx^M^::GFP*/*Tb* |  |
| 12C, D | *Yp1::GFP*/*CyO* |  |
| 12E, F, H, I, J | *c587-Gal4/Y; tub-Gal80^ts^/+; UAS-traF*^Δ^*^T2AGFP^/+* | *c587^ts^ >traF*^Δ^*^T2AGFP^* |
| 12G, H, I, J | *c587-Gal4/Y; tub-Gal80^ts^/UAS-zld; UAS-traF*^Δ^*^T2AGFP^/+* | *c587^ts^>traF*^Δ^*^T2AGFP^; zld* |
| **Supplementary Figures** | | |
| S3A | *w/Y; +/+; dpp-Gal4, UAS-GFP/+* | *dpp>+* |
| S3B | *w/Y; +/+; dpp-Gal4, UAS-GFP/UAS-zld-RNAi* | *dpp>zld-i* |
| S3C, D, G, H, I | *w/Y; tj-Gal4/+; +/+* | *tj>+* |
| S3E, G, I | *w/Y; tj-Gal4/+; +/UAS-zld-RNAi* | *tj>zld-i* |
| S3F, H | *w/Y; tj-Gal4/+; UAS-zld/+* | *tj>zld* |
| S3J-L | *sfGFP-Zld/Y* |  |
| S3N-P | *mNeonGreen-Zld/Y* |  |
| S3M | *sfGFP-Zld/Y; chinmo^ST^/+* | *sfGFP-Zld/Y*; *chinmo^ST^/+* |
| S3M | *FM7/Y*; *chinmo^ST^/chinmo^ST^* | *FM7/Y*; *chinmo^ST^/chinmo^ST^* |
| S3M | *sfGFP-Zld/Y*; *chinmo^ST^/chinmo^ST^* | *sfGFP-Zld/Y*; *chinmo^ST^/chinmo^ST^* |
| S3Q | *FM7/Y; tj-Gal4/+; +/+* | *FM7/Y; tj>+* |
| S3Q | *FM7/Y; tj-Gal4/+; UAS-dcr-2/UAS-chinmo-RNAi* | *FM7/Y; tj>chinmo-i* |
| S3Q | *mNeonGreen-Zld/Y; tj-Gal4/+; UAS-dcr-2/UAS-chinmo-RNAi* | *mNG-Zld/Y; tj>chinmo-i* |
|  |  |  |
| S4A | *Oregon-R* | WT adult ovary |
| S4B | *Oregon-R* | WT larval ovary |
| S4C | *Oregon-R* | WT larval testis |
| S4D | *Oregon-R* | WT larval VNC |
| S4E | *c587-Gal4/w; tub-Gal80^ts^/+; +/UAS-zld-RNAi* | *c587^ts^>zld-i* |
|  |  |  |
| S5A, C | *c587-Gal4/Y; +/+* | *c587>+* |
| S5B, C | *c587-Gal4/Y; +/UAS-Dcr-1-RNAi* | *c587>Dcr-1-i* |
| S5D, F, G | *Oregon-R* | WT |
| S5E, F, G | *w, miR-283KO/Y* | *miR-283KO* |
|  |  |  |
| S6A, D | *w/Y; tj-Gal4/+; +/+* | *tj>+* |
| S6B, D | *w/Y; tj-Gal4/+; UAS-Ir93a-RNAi* | *tj>Ir93a-i* |
| S6C, D | *w/Y; tj-Gal4/+; UAS-Gmap-RNAi* | *tj>Gmap-i* |
| S7A, C | *w/w; tj-Gal4/+; +/+* | *tj>+* |
| S7B, C | *w/w; tj-Gal4/UAS-qkr58E-2-RNAi #1; +/+* | *tj>qkr58E-2-i #1* |
| S7C | *w/w; tj-Gal4/UAS-qkr58E-2-RNAi #2; +/+* | *tj>qkr58E-2-i #2* |
| S7D, F | *c587-Gal4/Y; +/+; +/+* | *c587>+* |
| S7E, F | *c587-Gal4/Y; +/UAS-qkr58E-2^G3095^* | *c587>qkr58E-2^G3095^* |
|  |  |  |
| S8A, C | *w/w; tj-Gal4/+; UAS-traF*^Δ^*^T2AGFP^/+* | *tj>traF*^Δ^*^T2AGFP^* |
| S8B | *w/w; tj-Gal4/UAS-qkr58E-2-RNAi; UAS-traF*^Δ^*^T2AGFP^/+* | *tj>traF*^Δ^*^T2AGFP^; qkr58E-2-i #1* |
| S8C | *w/w; tj-Gal4/UAS-qkr58E-2-RNAi #1; UAS-traF*^Δ^*^T2AGFP^/+* | *tj>traF*^Δ^*^T2AGFP^; qkr58E-2-i #1* |
| S8C | *w/w; tj-Gal4/UAS-qkr58E-2-RNAi #2; UAS-traF*^Δ^*^T2AGFP^/+* | *tj>traF*^Δ^*^T2AGFP^; qkr58E-2-i #2* |
| S8C | *c587-Gal4/w; +/+; UAS-traF*^Δ^*^T2AGFP^/+* | *c587>traF*^Δ^*^T2AGFP^* |
| S8C | *c587-Gal4/w; +/UAS-qkr58E-2-RNAi #1; UAS-traF*^Δ^*^T2AGFP^/+* | *c587>traF*^Δ^*^T2AGFP^; qkr58E-2-i #1* |
| S8C | *c587-Gal4/w; +/UAS-qkr58E-2-RNAi #2; UAS-traF*^Δ^*^T2AGFP^/+* | *c587>traF*^Δ^*^T2AGFP^; qkr58E-2-i #2* |
| S8D, G | *w/w; tj-Gal4/+; +/+* | *tj>+* |
| S8E | *w/w; tj-Gal4/UAS-qkr58E-2-RNAi; +/+* | *tj>qkr58E-2-i* |
| S8F | *w/w; tj-Gal4, tub-Gal80^ts^/UAS-dsx-RNAi; UAS-Dcr-2/+* | *tj^ts^>dsx-i* |
| S8G | *w/w; tj-Gal4/UAS-qkr58E-2-RNAi#1; +/+* | *tj>qkr58E-2-i #1* |
| S8G | *w/w; tj-Gal4/UAS-qkr58E-2-RNAi#2; +/+* | *tj>qkr58E-2-i #2* |
| S8G | *w/w; tj-Gal4, tub-Gal80^ts^/UAS-dsx-RNAi#1; UAS-Dcr-2/+* | *tj^ts^>dsx-i #1* |
| S8G | *w/w; tj-Gal4, tub-Gal80^ts^/UAS-dsx-RNAi#2; UAS-Dcr-2/+* | *tj^ts^>dsx-i #2* |
|  |  |  |
| S9A, C | *w/Y; tj-Gal4/+; +/+* | *tj>+* |
| S9B, C | *w/Y; tj-Gal4/UAS-qkr58E-2-i; +/+* | *tj>qkr58E-2-i #1* |
|  |  |  |
| S10A, D | *c587-Gal4/Y; tub-Gal80^ts^/+* | *c587^ts^>+* |
| S10B, D | *c587-Gal4/Y; tub-Gal80^ts^/+; +/UAS-tra^F^* | *c587^ts^>tra^F^* |
| S10C, D | *c587-Gal4/Y; tub-Gal80^ts^/+; +/UAS-dsx^F^* | *c587^ts^>dsx^F^* |
