# Supplementary material for: Post-transcriptional suppression of the pioneer factor Zelda protects the adult Drosophila testis from activation of the ovary program
Source: PLoS Biol. 2025 Dec 18;23(12):e3003535. doi: 10.1371/journal.pbio.3003535 (PMC12714197; doi:10.1371/journal.pbio.3003535)
Supplement: S3 Table — The table additionally includes miRNA initiator probe sequences, target miRNAs, amplifier sets, probe lengths, and associated DNA oligonucleotide sequences. (DOCX) [file pbio.3003535.s014.docx]

| **S3 Table: Information for HCR-FISH** | | | |
| --- | --- | --- | --- |
| HCR probe of *qkr58E*-2 Molecular Instruments, Inc. LOT: PRJ993 | | | |
| HCR probe of *zld* Molecular Instruments, Inc. LOT: PRJ994 | | | |
| HCR probe of *chinmo* Molecular Instruments, Inc LOT: PRJ995 | | | |
| **miR probe sequences for HCR-FISH** | | | |
| **miR**  **target** | **Amplifier Set** | **Length** | **DNA oligonucleotide sequences** |
|  |  |  |  |
| **miR** |  |  | **Initiator Probe** |
| *miR-1011* | 1 | 64 | *CCTCGTAAATCCTCATCAATCATCCAGTAAACCGCCatataCTGCGAGCGATTTGAACCAATAA* |
| *miR-263a* | 1 | 65 | *CCTCGTAAATCCTCATCAATCATCCAGTAAACCGCCatataCCCGTGAATTCTTCCAGTGCCATT* |
| *miR-283* | 1 | 63 | *CCTCGTAAATCCTCATCAATCATCCAGTAAACCGCCatataCCAGAATTACCAGCTGATATTT* |
