# Supplementary material for: Post-transcriptional suppression of the pioneer factor Zelda protects the adult Drosophila testis from activation of the ovary program
Source: PLoS Biol. 2025 Dec 18;23(12):e3003535. doi: 10.1371/journal.pbio.3003535 (PMC12714197; doi:10.1371/journal.pbio.3003535)
Supplement: Attachment — Submitted filename: Harsh Response to Reviewers FINAL.pdf [file pbio.3003535.s018.pdf]

We thank the reviewers for their insightful and helpful comments. To address these, we have added new data documenting: (1) that Dcr-1 and multiple miRs in male somatic gonadal cell promotes male identity and prevents feminization; (2) multiple miRs repress *zld* mRNA in these cells; (3) Ecdysone receptor (EcR) mediates sex reversal downstream of Zld-overexpressing in male somatic gonadal cells. The number of figures has increased from 7 in the original manuscript to 13 in the revised one. The increase in the number of figures is due in part to accommodation of new data:

- Fig 2 about Dcr-1-depleted testes undergoing feminization
- Fig. 3 about significant increase in *zld* transcripts in testes mutant for *miR-1011*, *miR-263a* or *miR-283*
- Fig. 4 about increased Zld protein and feminization in double and triple miR mutants
- Fig. 11 about the role of EcR in repressing Chinmo downstream of Zld.

The increase in the number of figures is also due to our re-organization the some original figures, which had become large and exceeded the maximum height and width for *PLoS Biology.* We also added a new figure in Supplement Information (S2 Fig about the gene ontology for genes with Zld binding sites). We added a new data file (S1 Data) that contains all of the individual numerical values supporting all figures. We made many editorial changes to the text to response to the reviewers' comments and to format the manuscript for *PLoS Biology* (see manuscript with tracking changes). These changes have greatly improved the manuscript, which we hope is now acceptable for publication in *PLoS Biology*. Below is a point-by-point response to the reviewers' comments.

----------------------------------

Reviewers' comments

Rev. 1:
In general, I found the paper very informative and well written. It fills an important gap in our understanding of how sex reversal can occur in fully differentiated adult organs, so it should be of interest to a wide audience. The experiments are carefully conducted and well described. The use of multiple experimental approaches and well-chosen controls gives one strong confidence in the results. The writing is straightforward, and the figures are clear and well organized. I also like how the data are divided between the main text and the supplement. All in all, I think this is an excellent manuscript and I only have a few comments.

**Response**: We are grateful to the reviewer for this favorable synopsis of our work.

Introduction
The authors never explain that chinmo is not expressed in the normal ovary. I understand that

this background is well established, but without this piece of data, the logic doesn't make any sense, so it's worth saying explicitly.

**Response**: We thank the reviewer for pointing this out. We have added this sentence to the Introduction "Adult ovarian FSCs, on the other hand, do not express Chinmo or $Dsx^M$ (14, 15, 32)" in **lines 75-76.**

This is nitpicking, but in Lines 64-69, please use more careful phrasing. Mammals and flies are not "species"; and there are flies (such as Musca and Calliphora) that do not require a match between somatic and germline sex.

**Response**: We have changed the text to "In gonads, sex-specific somatic identity is essential for fertility and reproduction. In many mammalian species and in fly species such as *Drosophila melanogaster*, sex-specific somatic cells are required to promote the differentiation of sex-specific gametes and to impart sex identity to germline cells (25, 26). In these species, the production of gametes requires that the sex of the germline and the sex of the soma are the same." **Lines 63-67.**

Results
There's one set of results that I don't know what to make of. One of the central ideas in this paper is that (1) increased abundance of the Zld protein is the key mechanism by which the loss of chinmo induces testis feminization and (2) the effect of chinmo on Zld levels is mediated by the miRNAs miR283, miR1011, and miR263a. However, loss of chinmo results in the feminization of the testis, while miRNA loss does not (lines 205-206). This is despite the fact that, as far as I can tell, the changes in Zld levels are comparable. Zld expression appears to be increased roughly 3.5-4.5x in chinmo mutants and RNAi KO, 2.5x in miR283 KO, 6x in miR1011 KO, and 4-5x in the miR263a sponge (Fig 2). How do the authors explain this discrepancy? Do they believe that the increase in Zld levels that occurs following single miRNA loss is not as strong as what's seen in chinmo KO? Or is there some parallel mechanism through which chinmo acts independently of Zld? An obvious experiment would be to test double and triple miRNA mutants, though I imagine this experiment is technically difficult. Did the authors attempt it? A related observation is that a ~3-3.5x increase in Zld levels is seen in Dcr-1 KO (Supplement Fig 4). Does that result in testis feminization? The authors' model would seem to predict that it should, but I could not find any data on that. Anyway, some discussion of the discrepancies between the chinmo and miRNA phenotypes, despite the fact that both derepress Zld, would be welcome.

**Response**: We thank the Reviewer for this valuable suggestion and have performed the following experiments:

1. To address the concern about whether somatic depletion of Dcr-1 results in feminization, we have depleted Dcr-1 from somatic gonadal cells of the testes (1) using a stronger Gal4 driver (*c587-Gal4* instead of the original *tj-Gal4*) and (2) assessing feminization (Fas3-positive cells outside of the niche) at later time points. We used two distinct Dcr-1 RNA-interference transgenes. At 25 days post-induction of RNA interference, we found that Zld protein expression increased 12-fold and 6-fold and that feminization occurred in

52% and 25% of testes in Dcr-1-i#1 and Dcr-1-i#2, respectively. These results are presented in Fig 2D-G and S5A-C Fig. The corresponding text has been added to the revised manuscript in **lines 188-199**. We conclude that loss of the key enzyme in generating mature miRNAs results in significant feminization of XY somatic gonadal cells.

2. To address the role of miRs in regulating *zld* mRNA, we used HCR-FISH to measure *zld* transcript levels in somatic cells of single *miR-1011KO* and *miR-283KO*, which are homozygous viable, and a miR-sponge (SP) for *miR-263a*, whose genetic mutation is homozygous lethal. We found that *zld* transcripts increased 1.5-fold in *miR-1011KO*, 1.72-fold in *miR-263aSP*, and 1.5-fold in *miR-283KO* mutants. These new data are included in Fig 3N-R and in **lines 219-223**. We conclude that *miR-1011*, *miR-263a* and *miR-283* cause the repression of *zld* mRNA in WT XY somatic gonadal cells.

3. To address the role of double and triple miRNA mutants in feminization, we analyzed single *miR-1011KO* and *miR-283KO* and a miR-sponge for *miR-263a*. For double and triple mutants, we used combination of miR-sponges because of lethality prior to adulthood of stocks combining genetic mutants with miR sponges. For *miR-1011KO*, we analyzed testes at 25 days post-eclosion. For miR sponge experiments, we used the stronger *c587-Gal4* driver and assessed testes at 25 days post-induction. In testes from *miR-1011KO* at 25 days, we observed a 9-fold increase in Zld protein and 25% feminization of testes. In testes somatically depleted for *miR-263a*, we observed a 9-fold increase in Zld protein and 20% feminization. In *miR-283KO* testes, we observed a 3-fold increase in Zld protein but no feminization. In the double and triple mutants, any combination that included *miR-263a* sponge, 20% of testes were feminized which is not different from the single *miR-263a* sponge alone. These results suggest that the feminization observed in double and triple miR sponges is due to the *miR-263a* sponge. Somatic over-expression of single miR sponges for *miR-1011* or *mir-283* did not cause feminization. These new data are included in Fig 4 of the revised manuscript, **lines 228-253**. We conclude that *miR-1011* and *miR-263a* maintain male somatic sex identity by inhibiting *zld* mRNA.

Based on our new data, there is no longer a discrepancy between loss of *chinmo* and loss of miR production. However, the fact the feminization of single miRNA mutants (25% for *miR-1011KO* and 20% for *miR-263a* sponge) is not as large as that seen for somatic *chinmo* loss (100% feminization) suggests that there are either additional miRs that repress *zld* mRNA or that there are other mechanisms to inhibit *zld* mRNA. Unraveling these possibilities is of interest to us but is beyond the scope of this manuscript.

In response to the reviewer's comment about whether there are "parallel mechanism through which chinmo acts independently of Zld", we are not certain about their existence. One important result supporting the idea that feminization downstream of chinmo acts largely through Zld is that the hypomorphic alleles *sfGFP-zld* and *mNG-zld* significantly repress feminization (87% for the former and 80% for the latter, in S3M,Q Fig) in **lines 166-170**. This is the strongest repression of feminization that we have ever

seen.

Discussion

Prolonged Zld expression reduces chinmo transcription. EcR is higher in the ovary than testis, and is upregulated in chinmo mutants. Ectopic Zld causes higher EcR expression. An obvious hypothesis is that EcR mediates the long-term effect of Zld on chinmo expression. As the authors say in the Discussion section, the EcR story is complicated, and some results are contradictory. Nevertheless, it would be nice to discuss the evidence for and against this hypothesis directly, since it could have substantial explanatory power. At the moment, that section of the Discussion is the least clear (to me, anyway).

**Response:** To expand our original results, we directly tested the model that EcR (upregulated by Zld) leads to loss of Chinmo. To this end, we depleted *EcR* in Zld-overexpressing somatic cells of the testis (genotype: $c587^{ts}$ > *zld* + *EcR-RNAi*). We found that 33% of these testes displayed Chinmo loss and feminization. By contrast, 100% of testes somatically overexpressing Zld but not depleted for EcR displayed Chinmo loss and feminization. These results further strengthen our model that the downregulation of Chinmo seen in Zld-overexpressing somatic cells is caused by EcR. These new data are included in Fig 11, **lines 373-379**.

In terms of discussing the evidence for and against EcR mediating the long-term effect of Zld on chinmo expression, we have written a paragraph in the Discussion of the revised manuscript per this suggestion, **lines 426-441** and pasted below:

Our results raise the possibility that EcR directly silences the chinmo gene. If true, we would expect to observe EcR binding directly to the chinmo locus. This has not yet been reported in Zld-overexpressing male somatic cells. In the larval wing disc, downregulation of chinmo during normal development correlated with an increase in EcR, however EcR did not bind the chinmo locus, suggesting an indirect effect (73). EcR may silence the chinmo gene in male somatic gonadal cells. In support of this model, the EcR target Broad (Br) and Chinmo are reciprocal antagonists during development, possibly at the level of transcription (74, 75); however adult CySCs already express Br (71, 72), so this mechanism is unlikely. Another possibility is that EcR promotes expression of let-7 miRNA, which then downregulates chinmo-RH isoform that has a ~6 kb extension of the 3' UTR; this type of regulation of chinmo has been reported in mushroom body lineage (76). However, HCR-FISH revealed that the chinmo-RH isoform is not expressed in WT somatic cells of the testis (77), making this model unlikely. Finally, it is possible that EcR represses chinmo in male somatic gonadal cells through an unknown mechanism; in the larval neuroepithelium, the mechanism by which EcR downregulates Chinmo is also unknown (63). Future work using CUT&RUN and ATAC-seq will be needed to determine how EcR represses the chinmo gene in somatic cells of the testis.

My other point is more speculative. The authors present zld as having a somewhat specific function in female sexual differentiation. But another way to look at it is that zld, as a pioneer TF, may have a very generic role in transdifferentiation and cell fate reassignment (in addition to its "normal" roles in early development). In this case, zld is not expressed or required for normal ovary development in XX flies - it only seems to be involved in transdifferentiation of testis into ovary in mutant XY flies. In another context, zld is dispensable for normal wing development, but is required for cell fate respecification during wing disc regeneration

(https://urldefense.proofpoint.com/v2/url?u=https-
3A__pubmed.ncbi.nlm.nih.gov_38854062_&d=DwIBaQ&c=slrrB7dE8n7gBJbeO0g-
IQ&r=KgiGQ6wgd_t1IHvYuF63bDFsYGY79_-OCUjkfW9x37g&m=hvaiMugQCn_53X6ofjR-
cO31dO6e9GeVUdeOpfFVxEnPn_65Z2o2MIDrYEMqn9ov&s=5xtE2Au9vTQNnXVOJyovpN
kItZZCDx3qqIKbKlM4LHc&e= ). It can also reprogram partially differentiated neuroblasts back toward a neural stem cell identity (https://urldefense.proofpoint.com/v2/url?u=https-
3A__pubmed.ncbi.nlm.nih.gov_34887421_&d=DwIBaQ&c=slrrB7dE8n7gBJbeO0g-
IQ&r=KgiGQ6wgd_t1IHvYuF63bDFsYGY79_-OCUjkfW9x37g&m=hvaiMugQCn_53X6ofjR-
cO31dO6e9GeVUdeOpfFVxEnPn_65Z2o2MIDrYEMqn9ov&s=40ET2Gxp9Wt9zy2mMCmrPy
ufI3xt0jNur20tBzcvslQ&e= ). So, is it possible that zld does not induce sex reversal, but rather permits cell fate reprogramming more generally by allowing reactivation of previously silenced genes? In this case, there are almost 100 genes that are downstream of chinmo and have Zld binding sites, but the effect of chinmo on the splicing of tra (and thus feminization) seems to be largely facilitated by just one, qkr58E-2. Is it possible that ectopic expression of Zld simply opens up a random subset of its target genes, and that the instructive role in sex reversal is played by a different upstream regulator of qkr58E-2? Again, this is pure speculation. I am not suggesting that this alternative interpretation is correct, and I am not asking for an experimental test of this idea. I'm just bringing it up as a possibility. And perhaps it's worth considering zld functions in other contexts in the Discussion?

**Response**: The reviewer raises an interesting hypothesis about whether *zld* permits cell fate reprogramming and not sex reversal *per se*. We agree that Zld may have a broad role in activating a random set of its target genes that were previously silenced in the cell of interest. We have added text to the Discussion of the revised manuscript (**lines 471-480**) addressing this line of speculation and pasted here:

"While we conclude that Zld is required for sex reversal, it is possible that Zld plays a generic role in cell fate reprogramming and not in sex reversal per se. For example, Zld permits cell fate reprogramming in diverse contexts, including wing disc regeneration where zld is dispensable for normal development of the wing disc but essential for its regeneration (81), and in the nervous system where Zld reverts partially differentiated neuroblasts to a stem cell–like state (82). These findings, together with our results, raise the possibility that Zld acts more broadly by opening chromatin and reactivating silenced genes or by randomly activating a set of its target genes. In our case, feminization occurs from the activation of specific Zld targets *qkr58E-2* and *EcR*. Future studies using chromatin accessibility assays such as ATAC-seq will be essential to delineate the precise repertoire of Zld target genes in our model system."

Regarding the issue of whether Zld opens the *qkr58E-2* locus and then another transcription factor upregulates *qkr58E*-2 mRNA, this is possible. For this model to be correct, the *qkr58E-2* gene would need to be silenced in male somatic gonadal cells. In support of this, our HCR-FISH results show that *qkr58E-2* mRNA is very low in male somatic gonadal cells (Fig 7D, E, H). However, we do not know the state of chromatin in WT, *chinmo*-deficient, or Zld-overexpressing CySCs. To formally test the reviewer's model, we would need ATAC-seq analyses from testes of these genotypes, data sets which we do not have. It would be interesting to assess whether *qkr58E*-2 is upregulated in *chinmo*-deficient somatic cells by transcription

factors that are already present in male somatic gonadal cells or whether the transcription factor is itself upregulated by loss of *chinmo*. However, we do not know which transcription factors (besides Zld, EcR and $Dsx^F$) are upregulated in *chinmo*-deficient CySCs. We think that it is unlikely that $Dsx^F$ promotes *qkr58E-2* mRNA because (1) $Dsx^F$ downstream of Qkr58E-2 in our model and Brian Oliver's lab reported that $Dsx^F$ DamID did not label the *qkr58E-2* locus in ovaries (Clough et al, Table S1 PMID: 25535918).

Rev. 2:
Review: Post-transcriptional suppression of the pioneer factor Zelda protects the adult testis from activation of the ovary program

Summary:
Bach and colleagues seek to investigate the role of Zelda in somatic sex identity of tests and sex reversal of testes. The male identity of the adult somatic stem cells (cyst cells) in the testis requires the transcription factor Chinmo and through loss of chinmo, the testes are feminized and acquire ovary programming. Here the authors investigated the differentially expressed genes in chinmo depleted testis and found that 30% of the genes contained Zelda binding sites. The authors showed that in WT testes, the Zelda protein is not present via confocal imaging. To further investigate the role of Zelda in male identity of the testes cyst cells, the author depleted both zelda and chinmo in the cyst cells. By Fas3 assay, the testes in chinmo depleted cells were feminized while there was significantly less feminization in the cells where they depleted both zelda and chinmo. The authors then discovered that the Zelda transcript levels in chinmo mutants were unchanged despite the fact that there is more Zelda protein. They investigated further to interrogate whether zld transcripts are repressed by miRNAs in WT cyst cells. Using candidate miRNAs they hypothesized that chinmo was responsible for expressing the miRNAs that regulated zld mRNA repression. They found three miRNAs that were reduced in the chinmo mutant background, and also corresponded to an increase in Zelda protein when each of the three miRNAs were mutated. However, testes were not feminized in the single miRNA mutants so there is some redundancy. To test whether Zelda is sufficient to trigger feminization, the authors monitored the alternative splicing product TraF only present in WT ovarian FCs. The authors showed that ectopic expression of zld in cyst cells induced TraF splicing however this did not reach levels in WT ovarian FCs. The authors next looked at how Zelda might be impacting the splicing of TraF and found that in chinmo mutants, transcript levels of an RBP (Qkr58E-2) was misregulated.

This is a strong manuscript with high quality immunostaining and quantification. It addresses an important question of how gonads maintain sexual differentiation and avoid inappropriately switching sexes. The authors identified a novel microRNA pathway that is used to repress the induction of Zelda and used both antibody staining and tagged Zelda as tools to address their hypothesis.

**Response:** We are grateful to the reviewer for this positive assessment.

Questions:

The authors note that 30% of the target genes they identify have TAGteam sites and therefore might be regulated by Zelda. However, the presence of a binding site alone is not highly predictive of binding.

Can the authors mine available multi-omic data from the Fly Cell Atlas to determine if these specific TAGteam sites change in their accessibility status in different tissues or in males versus females?

Additional characterization of these genes in terms of Gene ontology analysis would be helpful.

**Response**: We thank the Reviewer for this thoughtful suggestion. We agree that the presence of a TAGteam site alone is not sufficient to infer Zld binding or functional regulation. The Fly Cell Atlas provides high-quality single-cell RNA-seq datasets across tissues and sexes; however, these data capture gene expression but neither chromatin accessibility nor transcription factor binding. As such, they cannot directly inform whether specific TAGteam sites are bound by Zld or whether their accessibility changes across tissues or between males and females.

In response to the reviewer's comment about gene ontology, we performed analysis (using DAVID), which revealed that the most significantly enriched gene ontology terms among putative Zld target genes were related to actin organization, epithelial morphogenesis, and cytoskeleton organization, to name a few, consistent with the gain of ovarian somatic gene expression. We have added this information to **lines 133-137** and new supplemental S2 Fig. We conclude that Zld target genes are enriched in processes that are essential for female somatic gonadal cells.

Can the authors measure zelda RNA levels by qPCR or FISH to show that the transcript is being regulated by the microRNAs identified because the current studies rely only on immunostaining? Therefore, it is possible that the microRNAs are functioning indirectly through targeting of another factor that regulates Zelda protein levels.

**Response:** We have already discussed this experiment in response to R1. Please see that comment. To briefly summarize here, we measured *zld* transcript levels in somatic cells of miRNA mutants and miR sponges using HCR-FISH. *zld* transcripts increased 1.5-fold in *miR-1011KO*, 1.72-fold in *miR-263aSP*, and 1.5-fold in *miR-283KO* mutants, consistent with regulation by these miRNAs. These new data are included in Fig 3N-R and discussed in the revised manuscript on **lines 219-223**.

We agree with the reviewer that it is possible that *miR-1011* and *miR-263a* are functioning indirectly through targeting of another factor that regulates Zld protein levels. Nevertheless, our work has identified these miRNAs as being important for suppressing *zld* mRNA in male somatic gonadal stem cells.

The Qkr58E-2 experiments are a little counterintuitive: Even with more Zelda, there is no

feminization without the RBP, so wouldn't Qrk58E-2 be more important for maintaining the "male identity" and preventing female identity of the somatic cells.

**Response**: We respectfully disagree with the reviewer's interpretation of our work. As we understand it, the reviewer's comment states that Qkr58E-2 is important for maintaining male identity. We believe that our results support our model that Qkr58E-2 is important for feminization, and we would like to highlight the following points to support our conclusion.

1. HCR-FISH results show that *qkr58E-2* transcripts are female-enriched and are 2.5-fold higher levels in somatic ovary compared to the somatic testis.
2. We validated two independent *qkr58E-2* RNAi lines using HCR-FISH.
3. Using these validated RNAi transgenic lines, we showed that Qkr58E-2 is a *bona fide* regulator of *tra* pre-mRNA alternative splicing in ovarian follicle cells; depleting *qkr58E-2* in ovarian somatic cells reduced both $Tra^F$ and Fas3 expression in 36% of ovaries in addition to causing oogenetic defects. By contrast, depletion of *qkr58E-2* in adult somatic testes had no discernable phenotype.
4. Overexpression of *qkr58E-2* in male somatic cells of testes is sufficient to trigger ectopic expression of female-specific factor $Tra^F$ and feminization (i.e., epithelial Fas3 expression in non-niche cells).

Taken together, these findings support our model that the Zld target gene *qkr58E-2* facilitates activation of female program in chromosomally male cells.

Zelda intensity is increased to the same amount in the miRNA mutant as in Figure 1 BUT there is no feminization. How do the authors reconcile this?

**Response**: R1 asked a similar set of questions. Please see our response to R1. Briefly, our new results show that depletion of the enzyme that produces mature miRNAs (i.e., Dcr-1) leads to significant feminization of male somatic gonadal cells, as does loss of *miR-1011* or depletion of *miR-263a*.

Minor Comments on text:
Ending of the introduction jumps around a little, hard to follow logic after mentioning the targets of Zelda in the embryo

**Response:** We have re-written the last paragraph of the Introduction, which we believe is now more linear and easier to follow (**lines 98-122**).

Why do RNAi and the KO

**Response**: We don't understand what the reviewer is requesting.

Miss expressing vs overexpressing is confusing

**Response**: We have replaced "misexpression" with "overexpression" throughout the revised manuscript.

The phrase "Zelda is required for conversion of adult male gonadal somatic cells into their

female counterparts" is confusing:
Because feminizing is NOT a normal phenomenon it would be good to rephrase: Zelda is important for maintaining MALE somatic stem cell identity and preventing feminization?

**Response:** We respectfully disagree. We believe that our results are the opposite of what the reviewer is suggesting. Our results demonstrate that *zld* mRNA must be suppressed by miRNAs to prevent ectopic expression of Zld protein, which can lead to feminization of XY somatic cells. Therefore, we submit that the ***suppression*** of Zld is important for maintaining male somatic stem cell identity.

Move "sustained ectopic Zld suppresses…" paragraph after the RBP stuff and right before the "ectopic EcR expression…" paragraph

**Response:** We thank the reviewer for the suggestion. We have done this, now presented in **lines 333-345**.

The model figure is confusing with different parts greyed out. It would be better to use an arrow with a line at the end to show repression.

**Response:** We agree with the reviewer and have made the suggested modifications to the model (Fig 13).

Rev. 3:
In their study "Post-transcriptional suppression of the pioneer factor Zelda protects the adult testis from activation of the ovary program", Harsh and Bach et al use genetic strategies to elucidate a multistep regulatory program that controls sexual identity.

Their stated highlights are:
* zld mRNA is repressed by microRNAs in XY somatic gonadal cells
* Zld is upregulated in and required for sex reversal of XY chinmo-/- cells
* Zld induces Qkr58E-2 and EcR, which cause TraF and female-biased transcription
* Zld feminizes XY adipose cells by inducing TraF and downregulating Chinmo

I think these conclusions supported by their data and are both interesting and thorough. In particular, the imaging data are compelling and beautiful, and new regulatory links are uncovered. On the miRNA side, they used both mutant (knockout or inhibitor) and misexpression studies to demonstrate phenotypic impacts of several miRNAs to suppress zelda in testis soma. One note is that it is fast becoming a standard to engineer mutations of miRNA binding sites as definitive proof of the biological impact of miRNA mediated regulation on specific target genes. This is due to the fact that miRNAs tend to have many targets, which might or might contribute to the phenotype of the miRNA mutant. However, since this study goes beyond simply focusing on zelda as a target of miRNAs, I think that it can be left to future

studies to generate mutations of miRNA binding sites in zelda.

Overall, I think they did a great job to elaborate a pathway upstream of zelda (miRNAs) and downstream of zelda (chinmo and Qkr58E-2), thus involving transcriptional and post-transcriptional effectors. I have only some small comments that I think would be easy to do.

**Response**: we appreciate the reviewer's enthusiastic evaluation of our study.

Major comments

1. Its very notable that multiple miRNAs had an effect either on expression of Zelda, or in testis cytology. This is interesting because many if not most miRNAs have relatively little effects, and if there are multiple functionally overlapping miRNAs, they might compensate for each other. This could be interpreted that Zelda is a very sensitive target of miRNAs. Given their in situs indicate co-expression of the miRNAs, this is a very testable scenario.

I might have missed if they did it, apologies if so, but I noted Fig 3 had single miRNA mutants, and there are tests with multiple miRNA-GOF, but not combinations of miRNA mutants. I think there is a strong expectation that combination miRNA mutants would yield strong effects. Since they have the reagents, I think its not hard to test at least some combinations.

**Response:** R1 asked a similar set of questions. Please see our response to R1.

Minor comments
1. I am curious about the miRNA in situs, since this is known to be a difficult technique. My understanding is that one can always get some signals by developing the reaction longer or by increasing gain etc. I am wondering how they determine that some miRNA in situ probes don't provide evidence of signal. This may be worth documenting in the supplement, and maybe comparing exposure times with real miRNA signals.

**Response:** For the miR HCR-FISH, samples from WT, somatic *chinmo*-mutant, and *miR* mutant (or miR sponge) were performed in parallel using a master mix of reagents and were imaged in parallel using the same confocal settings on the same day. We are confident about our results because we saw little or no signal in miR mutants or miR sponges. By contrast, we observed robust signal for each miR in WT samples. We have added a sentence to the Material and Methods section "WT, somatic *chinmo* depletion, and *miR* mutant (or miR sponge) samples were performed in parallel using a master mix of reagents and were imaged in parallel using the same confocal settings on the same day." on **lines 686-688**.

2. "Figure 2: zld is post-transcriptionally modified"
It is more appropriate to state that zelda is post-transcriptional regulated, rather than modified, as the latter implies the RNA is subject to an epitranscriptomic modification.

**Response:** We have changed the title of Fig 2 to "*zld* is post-transcriptionally regulated in *chinmo*-deficient somatic cells." in **line 756.**
